# Direct Biocatalytic Processes for CO_2_ Capture as a Green Tool to Produce Value-Added Chemicals

**DOI:** 10.3390/molecules28145520

**Published:** 2023-07-19

**Authors:** Rocio Villa, Susana Nieto, Antonio Donaire, Pedro Lozano

**Affiliations:** 1 Departamento de Bioquímica y Biología Molecular B e Inmunología, Facultad de Química, Universidad de Murcia, 30100 Murcia, Spain; rocio.villa@um.es (R.V.); susanani@um.es (S.N.); 2 Department of Biotechnology, Delft University of Technology, 2629 HZ Delft, The Netherlands; 3 Departamento de Química Inorgánica, Facultad de Química, Universidad de Murcia, 30100 Murcia, Spain

**Keywords:** carbonic anhydrase, formate dehydrogenase, carbon capture storage and its utilization, cofactor regeneration

## Abstract

Direct biocatalytic processes for CO_2_ capture and transformation in value-added chemicals may be considered a useful tool for reducing the concentration of this greenhouse gas in the atmosphere. Among the other enzymes, carbonic anhydrase (CA) and formate dehydrogenase (FDH) are two key biocatalysts suitable for this challenge, facilitating the uptake of carbon dioxide from the atmosphere in complementary ways. Carbonic anhydrases accelerate CO_2_ uptake by promoting its solubility in water in the form of hydrogen carbonate as the first step in converting the gas into a species widely used in carbon capture storage and its utilization processes (CCSU), particularly in carbonation and mineralization methods. On the other hand, formate dehydrogenases represent the biocatalytic machinery evolved by certain organisms to convert CO_2_ into enriched, reduced, and easily transportable hydrogen species, such as formic acid, via enzymatic cascade systems that obtain energy from chemical species, electrochemical sources, or light. Formic acid is the basis for fixing C_1_-carbon species to other, more reduced molecules. In this review, the state-of-the-art of both methods of CO_2_ uptake is assessed, highlighting the biotechnological approaches that have been developed using both enzymes.

## 1. Reducing Carbon Dioxide from the Air: The Challenge

One of the main challenges faced by humanity in the 21st century is climate change. Over the last two centuries, the temperature of the Earth’s crust has risen progressively and, since 1980, alarmingly, at a rate of 0.18 °C per decade. Indeed, last year’s average temperature was 1.04 °C higher than the median temperature in the period prior to 1880 [1]. Temperature elevation drives an increase in extreme weather evidenced by a series of well-known events (draughts, floodings, torrential downpours, etc.), the melting of large extensions of frozen water, with the subsequent ascent of the sea, changes in ecosystems with undefined outcomes and, in this sense, uncertainty on how these changes will affect our way of living and welfare, with estimations that are clearly detrimental [2]. Moreover, the acidification of seas and oceans is also a problem, with coral reef weakening already having been detected, as well as the low level of oxygen present in marine life [3,4].

At the end of the nineteenth century, S.A. Arrhenius quantified the contribution of “carbonic acid” (nowadays, carbon dioxide) to the greenhouse effect and was the first in indicating that “*The production of carbonic acid by the combustion of coal would therefore suffice to cover the loss of carbonic acid by weathering and by peat formation seven times over. Those are the two chief factors deciding the consumption of carbonic acid, and we thus recognize that the percentage of carbonic acid in the air must be increasing at a constant rate as long as the consumption of coal, petroleum, etc., is maintained at its present figure, and at a still more rapid rate if this consumption should continue to increase as it does now*”. He also concluded that this would lead to an increase in the temperature of Earth’s atmosphere [5,6]. Since then, a huge amount of evidence correlating both air CO_2_ concentration and global warming has accumulated [2,7]. Moreover, there is a direct relationship between human activity, carbon dioxide concentration, and climate change, that is, the anthropogenic origin of global warming is well established. Atmospheric CO_2_ concentration has increased from 280 ppm (year 1750) to 415 ppm (2021), this value being the highest concentration reached in the last three million years [8]. This carbon dioxide increase is essentially related to the emissions of this gas to the atmosphere as a consequence of the use of fossil fuels by humans [9]. Other gases, such as methane and nitrous oxide, also contribute to global warming, albeit to a minor extent (11 and 7%, respectively) [10]. The objective reached at the Paris Climate Agreement in 2015 to maintain an increase in overall temperature below 2.0 °C with respect to preindustrial levels has recently been revised in the sense that such increments should not exceed 1.5 °C [11].

In this scenario, any scientific strategy that allows for reducing the concentration of CO_2_ in the atmosphere is an object of interest, although the most relevant solution is to avoid burning fossil energy sources that release CO_2_, and to substitute them with other sustainable ones. Although more efficient and responsible use of fossil energy sources by society is also essential to contributing to decreasing CO_2_ emissions, the responsibility of capturing the excess CO_2_ already emitted is also inescapable.

**Figure 1:**
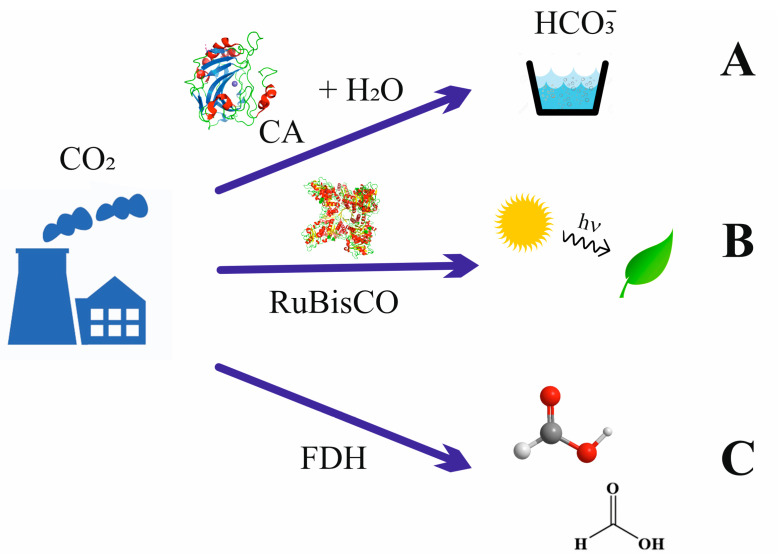
Three pathways to incorporating CO_2_ into soluble carbon forms by using enzymes. (**A**) Formation of hydrogen carbonate using carbonic anhydrase; (**B**) photosynthesis performed using RuBisCO protein; (**C**) reduction to achieve C_1_ in reduced form, formic acid, catalyzed using FDHs. Approaches A and C are the aims of the present work (see text for details).

Among other approaches, biocatalysts are useful tools for reducing the accumulation of atmospheric CO_2_ through either carbon capture and storage (CCS) or carbon capture and its utilization (CCU, Figure 1A). Both approaches are often applied together, known as carbon capture storage and utilization (CCSU) [12]. These methodologies require the passing of CO_2_ from gas to carbon solid and/or chemically reduced forms, a task that directly implies chemistry in all its fields.

**Figure 2:**
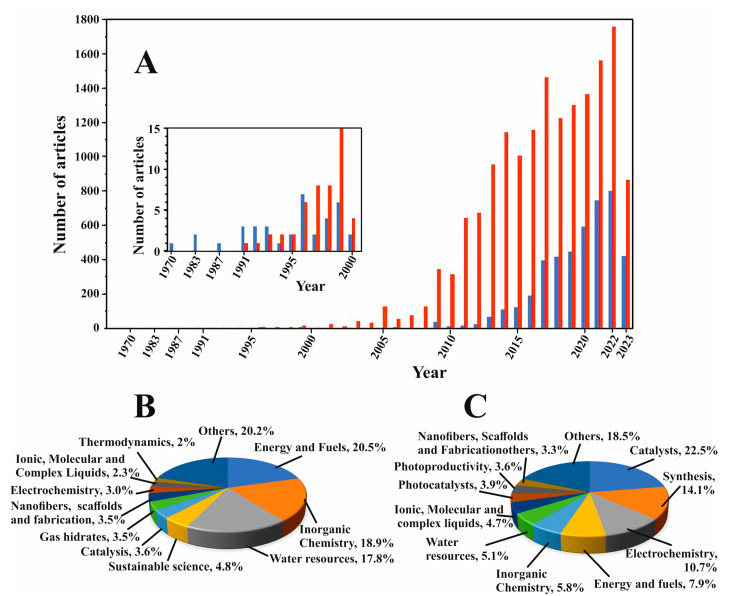
(**A**) Number of articles published per year related to carbon capture storage (red) and carbon capture utilization (blue); the insert is a vertical expansion, scale 0–15, by year, published in the last century (the year 2023 contains the number of articles at halfway through the year). (**B**,**C**) Percentage of articles related to CCS (**B**) and CCU (**C**) classified by research area. Source: compiled by the authors, based on the Web of Science database (https://clarivate.com), accessed on 2 July 2023.

Research in this field has exponentially increased in the last decade. Indeed, Figure 2A shows the number of articles published, directly or indirectly, that relate either to carbon storage or carbon utilization per year, while Figure 2B,C display the percentages of these articles classified by research area. As observed, research on carbon capture is especially intense in areas such as catalysts, synthesis, electrochemistry, and energy and fuels. Nowadays, there are two main approaches for CO_2_ transformation, namely biological and chemical transformations [13]. In turn, biological CO_2_ fixation can be photosynthetic or not photosynthetic, while the chemical uptake can be divided into hydrogenation, carboxylation, mineralization, chemical reduction, and photochemical reduction. Although hydrogenation is a well-established technology, nowadays, it is still mostly not green, as it is obtained from the cracking of fossil fuels. The sustainable synthesis and transport of hydrogen is indeed one of the main challenges related to energy sources. The main target of hydrogenation is the production of methanol (see Section 7.2), although other reduced molecules can also be obtained.

Carboxylation is the process of directly converting CO_2_ into organic value-added compounds. Organic carbonates and polymers are obtained through this process. An example is the production of non-isocyanate polyurethanes (NIPUs) from glycerol carbonates derivatives obtained through CO_2_ cycloaddition to glycidol moieties [14]. In this case, the authors designed a sustainable chemoenzymatic protocol for the synthesis of glycerol carbonate acrylate (GCA) and glycerol carbonate methacrylate (GCMA) from glycidol and CO_2_ by using ionic liquid (IL) technologies and enzymes, providing conversions of up to 100% under low-pressure values (1–10 bar). These methods are still in their first stages of development and hence a relatively novel field to work in.

Mineralization is described in Section 6.3 and is used in construction, for instance, for generating cement. This technique allows for the uptake of large quantities of CO_2_ for obtaining sustainable materials, although it is a highly consuming energy at a high scale. Chemical, electrochemical and photochemical reduction combined with enzymes is described below (see Section 7.2, Section 7.3 and Section 7.4).

The genuine physical and chemical properties of CO_2_ make it a molecule that cannot be easily captured or retained. Thus, CO_2_ is a nonpolar molecule that resides in a gaseous state under P and T standard conditions because of the weak van der Waals interactions established between the molecules themselves. In addition, due to its null polarity, CO_2_ has a low diffusion coefficient in polar solvents (1.26 × 10^−5^ cm^2^/s in water under standard conditions), while its solubility follows Henry’s law (76.5 mM in water at 0 °C and partial pressure of 1 atm) [15]. More importantly, its kinetics of capture by water, while increasing with pH, is extremely low at neutral or acidic pH values, behaving as a Brönsted acid according to Equations (1) and (2) (Scheme 1). The *K*_h_ value (Equation (2)) indicates that only a minimal fraction of CO_2_(aq) is present in an aqueous solution of carbonic acid (ca. 1/600 of the molecules at neutral pH values). Both hydrated carbon dioxide and carbonic acid behave as weak Brönsted acids because of the low deprotonation constants (see Equations (3) and (4)) [16].

(1) $${CO}_{2}\left(g\right)+H_{2}O\;⇄{CO}_{2}\left(aq\right)$$

(2) $${CO}_{2}\left(aq\right)+H_{2}O\;⇄H_{2}{CO}_{3}\to K_{h}=0.00159$$

(3) $${CO}_{2}\left(aq\right)+H_{2}O\;⇄{HCO}_{3}^{-}+H^{+}\to K_{a1}=4.3\times 10^{-7}\;M$$

(4) $${{HCO}_{3}^{-}}_{}+H_{2}O\;⇄{{CO}_{3}^{2-}+H^{+}}_{}\to K_{a2}=4.84\times 10^{-11}\;M$$

(5) $${{CO}_{2}^{}}_{}+NADH+H^{+}\;⇄{HCOOH+{NAD}^{+}}_{}$$

The equilibrium shown in Equation (3) is essential for the role of CO_2_ as a buffer between blood and cells, where the interconversion between CO_2_ and HCO_3_^−^ (hydro carbonate or bicarbonate) forms should be fast in living beings. Under neutral or weak basic conditions, the formation of bicarbonate anions is slow (the first order kinetic constant, *k*_1_, is of the order 10^−2^ s^−1^), while it increases in basic media [17]. Consequently, living organisms must have a biocatalyst that allows for fast exchange between acid and basic species. Carbon dioxide gas can be fixed either in aqueous soluble (hydrogen carbonate) or much more insoluble (carbonate) forms using only alkalization or using other chemical processes that incorporate carbon into value-added compounds. In nature, carbonic anhydrase (CA) catalyzes the CO_2_/hydrogen carbonate reaction (Equation (3)), enabling CO_2_ capture within reasonable times [17,18]. Thus, CA is used as a tool for incorporating CO_2_ as a hydrogen carbonate anion in soluble species in a multitude of chemical approaches [19,20]. In contrast, carbonates are usually highly insoluble. It follows that the use of alkaline reactants (Equation (4)) is the easiest and, consequently, main method for sequestering CO_2_ by forming the corresponding salts or their derivatives.

Alternatively, CO_2_ can be fixed to hydrogen-enriched energy forms via several natural mechanisms [21,22]. Although the most extended and productive mechanism in nature (green plants and algae) is photosynthesis, where ribulose-1,5-bisphosphate carboxylase-oxygenase (RuBisCo) converts CO_2_ and water into C_3_-carbohydrates by taking energy from light (Figure 1B) [22,23,24], here, the focus is on nonphotosynthetic enzymes. The conversion of CO_2_ into formate anion (C_1_ species, Equation (5), Scheme 1) is the simplest way in which nature captures CO_2_ into reduced, highly energetic molecules, and formate dehydrogenases (FDH) are the main actors in this performance (Figure 1C) [25,26,27,28,29].

**Figure 3:**
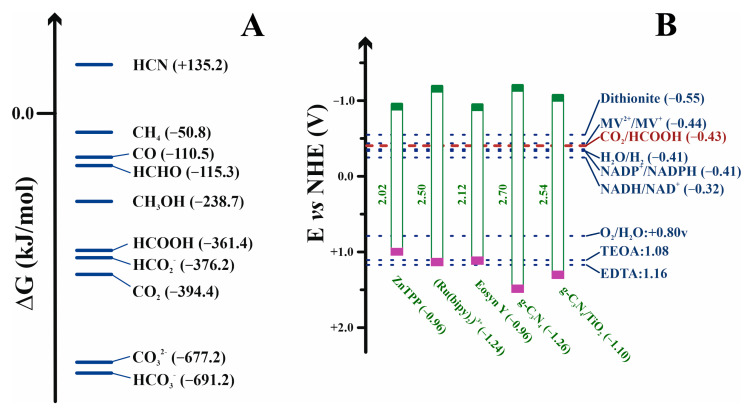
(**A**) Free formation energies of some C_1_ compounds under standard conditions. (**B**) Redox potentials vs. the normal hydrogen electrode (NHE) at pH 7 of some relevant compounds, photosynthesizers, and sacrificial agents (see Section 7.4) commented in the text [32,33]. The redox potential values are shown between parentheses. The HOMO and LUMO energy orbitals for some dyes are indicated in green and purple, respectively.

Formic acid, or its basic form formate, is a simple molecule for efficient hydrogen transport. Moreover, formate synthesis is the first step for obtaining other more complex and energetically enriched molecules [22,30]. Carbon dioxide and formic acid have similar formation energies (Figure 3A), with the most similar energetic states for C_1_-carbon forms. This converts formic acid as the easiest starting point for obtaining other C_1_ more reduced carbon species. Nevertheless, reaction 5, in the forward sense, is highly endergonic under physiological conditions. Indeed, the redox potential at pH 7 for CO_2_ reduction to formate is −430 mV [31], while that of NADH is only −320 mV (Figure 3B). More importantly, for the reasons discussed below, this first step is kinetically, energetically, and economically expensive, and it becomes the bottleneck for obtaining value-added products in CO_2_ regeneration. Several approaches for circumventing this issue are discussed below. However, other no less relevant problems arise when this reaction is performed for biotechnological purposes. FDHs from different organisms can solve these problems and incorporate formate anions into their biosynthetic routes. Thus, nature provides solutions to the previous difficulties. It is a researcher’s task to adjust these solutions at laboratory and industrial levels for the benefit of society.

In the following pages, the enzymes that participate in both processes of CO_2_ capture using CA (CCUS processes) and FDH (formate synthesis) are described. How these natural systems circumvent the thermodynamics and kinetics problems derived from reactions 3 and 5 are also discussed, paying special attention to the state-of-the-art concerning the biotechnological applications of these enzymes for CCS and CCU technologies, as well as for reducing CO_2_ to formic acid.

## 2. Carbonic Anhydrases: Efficient Devices for CO_2_ Uptake

### 2.1. Classification and Structure of Carbonic Anhydrases

Carbonic anhydrases (EC 4.2.1.1) are found in all living kingdoms [18,34,35,36]. They catalyze the reaction of interconversion between CO_2_ and HCO_3_^−^ (Equation (3)) in nature and are generally monomeric proteins with molecular weights roughly comprised between 30–50 kDa, depending on their class. CAs have been classified into: α-CAs (found in animal cells, algae, and eubacteria), β-CAs [37] (found in higher plants, microalgae, eubacteria, archaebacteria, and fungi), γ-CAs [37] (algae), δ-CA [38,39,40] (found in the marine diatom *Thalassiosira weissflogii*), ε-CAs [41] (found, for example, in the carboxysomal shell of *Halothiobacillus neapolitanus*), ζ-CA [42] (found in *Thalassiosira weissflogii),* η*-CA* [43] *(found in Plasmodium falciparum*), θ*-CA* [44], and ι-CA [45,46,47] (found in *Burkholderia territorii* and *Phaeodactylum tricornutum*, among other bacteria). CAs are zinc(II) enzymes, although some of them can contain other metal ions active in physiological roles: Cd(II) has been found in δ-CA, although its role is disputed [48,49]; Fe(II) is present in some γ-CAs growing under anaerobic conditions [50]; Co(II) has also been replaced in many α-CAs with excellent activities [51], although its original role has not been proven. Remarkably, a group of proteins called “COG4337” has been described as ι-CAs, and strikingly, those from the cyanobacterium *Anabaena* sp. PCC7120 and the chlorarachniophyte alga *Bigelowiella natans* display activity without any metal involved [52]. Table 1 lists the main features of some representative CAs.

**Figure 4:**
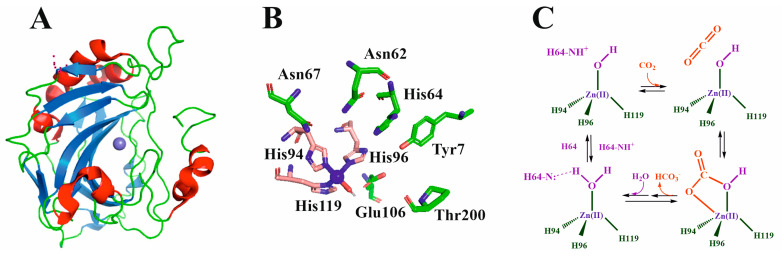
(**A**) Three-dimensional structure of human CA (5ZXW pdb code [68]). (**B**) Active center of the enzyme showing the most relevant amino acids (see text for details). (**C**) CA mechanism of action.

Mammalian CAs belong to the α-CA class. Depending on their location and primary sequences, several isoforms of human carbonic anhydrases (HCA) have been described, with HCAII being the most studied and best-characterized CA. The HCAII tertiary structure (Figure 4A) consists of a unique domain containing ten β-strands that twist to form a β-sheet (eight of them organized in an antiparallel arrangement and the other two in parallel) [68,69]. Surrounding these β-sheets, up to eight other α-helixes are located on the surface of the protein.

The structure of HCA is the most studied of all HCA isoforms, and its active site can be defined as a cone-shaped cleft, 15 Å deep, formed by a hydrophilic region (Tyr7, Asn62, His64, Asn67, Thr199, and Thr200) and a hydrophobic region (Val121, Val143, Leu198, Val207, and Trp209). Although the core of the active site in α-CAs is highly conserved, there is variability in the polarity and hydropathicity of its periphery [70]. The catalytic Zn(II) ion is located in a deep-centered slot that is accessible to the solvent. The Zn(II) ion is coordinated to three histidine residues (Figure 4B). While His94 and 96 are coordinated through their Nε2 imidazole nitrogen to the metal ion, His119 is bound through its Nδ1 imidazole nitrogen (HCAII numeration) [48]. A water molecule completes the tetrahedral coordination of Zn(II). A second coordination sphere is formed by amino acids that are not directly coordinated with the metal ion but are essential in the catalytic process. Through the formation of a network of hydrogen bonds, residues Tyr7, Asn62, His64, Asn67, Glu106, Thr199, and Thr200 stabilize the mediator species in such a way that the reaction can occur.

The HCA family includes a subclass of three noncatalytic isoforms (HCAs VIII, X, and XI) called CA-related proteins (CA-RPs), whose classification is based on their sequence. The noncatalytic behavior is due to the absence of one or more histidines that coordinate the Zn(II) ion of a catalytic HCA isoform. For instance, in HCA-RP VIII, the Zn-coordinating His94 (HCA II numbering) is replaced by an arginine (Arg116, according to HCA-RP VIII numbering). This residue avoids CO_2_ hydration in the first step of CA catalysis. [71]. Although the biological functions of CA-RPs have not been defined, these isoforms are of high interest in different scientific research fields. Recently, the X-ray crystal structure of only one HCA-RP (HCA-RP VIII) was determined [72]. HCA-RP VIII is expressed in the cerebellum [73] and has been identified as a binding partner for the inositol 1,4,5 triphosphate (IP3) receptor type [74]. It should be mentioned that the stability and structures of other HCAs such as HCA III or extracellular HCAs (i.e., IV, VI, IX and XIV) have not been as extensively studied as HCAs I and II. For instance, the bovine CA III showed a similar unfolding profile to that of HCA II, providing a molten globule intermediate and an unfolded state at a C_m_ of 2.6 M guanidinium chloride (GuHCl) concentration [75]. On the contrary, the isoforms VIII, X, and XI showed two distinct transitions, and their sensitivity to guanidinium chloride chemical denaturalization was higher than that of HCA II (C_m_ 0.4 M for HCA-RP VIII and 0.9 M for HCA II) [71].

### 2.2. CA Mechanism of Action

Carbonic anhydrase accelerates reaction 3 by more than six orders of magnitude (Table 1) with respect to its rate without the biocatalyst [18,76] (from ca. 3.6 × 10^−2^ M^−1^ s^−1^ to 1.0 × 10^6^ M^−1^ s^−1^ in the absence and presence of CA) [17], allowing for the reaction to take place under physiological conditions. Otherwise, CO_2_ cannot be assimilated as bicarbonate by living organisms.

The kinetic parameters of CA can be determined by measuring hydratase activity (Equation (3), Scheme 1). The method basically consists of saturating a buffered water solution (typically Tris.HCl 0.020 M, pH 8.3) kept in an ice bath with CO_2_ at a fixed high pH value, then adding CA, and measuring the time that the solution takes to reach a low pH value (generally ca. 6.3) because of the conversion of carbon dioxide to hydrogen carbonate anion that takes the capture of protons (Equation (3)) and hence lowers pH. The Wilbur–Anderson hydratase activity unit [77] is calculated as the ratio *((t_0_ − t)/t_0_)/(mg enzyme)*, where *t_0_* and *t* are the measured times that an indicator present in the solution shifts its color for the control (without CA) and the sample (in the presence of CA), respectively. This method provides a qualitative measurement of CA activity, but it is not strictly transferable from one set of experiments to another [78]. This method depends on the degree of saturation of CO_2_ (which in turn changes with temperature and the time at which CO_2_ is bubbled), the nature of the buffer, its concentration, and its initial pH value. Together, these units are roughly—not strictly—comparable. Most classes of CAs also exhibit esterase activity (δ-CA lacks esterase activity), whose measurement is more direct and contrastable [79]. This is generally performed by the hydrolysis of *p*-nitrophenyl acetate, which releases free *p*-nitrophenol, with maximum absorption at 400 nm, which is easily measurable [80]. Accurate experimental requirements for the latter experiments (concentration of the reactants, enzyme, and pH) are easily reproducible and should depend only on contrastable and exchangeable conditions.

The mechanism of action of CA has been deciphered [81] and basically consists of two stages (Figure 4C). In the first phase, a CO_2_ molecule, partially stabilized by interactions with groups of the enzyme active site, reaches the active center, and subsequently, the hydroxide group bound to the Zn(II) ion attacks the CO_2_ carbon atom (nucleophilic attack). Then, hydrogen carbonate is formed, and a water molecule replaces it through Zn(II) coordination. In the second step, a proton is transferred from the Zn(II)-coordinated water molecule to the solvent. This is the rate-limiting step. His64 is the amino acid responsible for accepting this proton, which is finally transferred to the bulk solvent. Due to this mechanism, at neutral/soft acid pH levels, the rate of CO_2_/HCO_3_^−^ conversion is enhanced by more than six orders of magnitude, making life possible. At pH values higher than 9.5, a direct hydroxide attack can form carbonate anions at rates comparable to those performed by CA at neutral pH values.

## 3. Formate Dehydrogenases: Natural Machines for Reducing CO_2_

The specific reduction in carbon dioxide converts it into formate/formic acid (Equation (5), Scheme 1). This C_1_ metabolism reaction occurs in hydrogenotrophic methanogens (*Euryarcheota*) and autotrophic acetogens (bacteria) and is carried out by the enzyme formate dehydrogenase (EC 1.17.1.9) [24,82,83,84,85]. Energy and high reduction power (i.e., a cofactor in the NAD(P)H form) are required to perform this process. In contrast, formate dehydrogenases also catalyze the backward reaction (5), oxidizing formate anions and obtaining energy from them. FDHs are divided into two main groups: nonmetal- or NAD-dependent FDHs, and metal-dependent FDHs. The structure, nature, and activity of these two FDH sets are completely different. More importantly, the ability of these two groups to reduce CO_2_ is manifestly diverse, as discussed below.

### 3.1. Metal-Independent/NAD-Dependent FDHs

**Figure 5:**
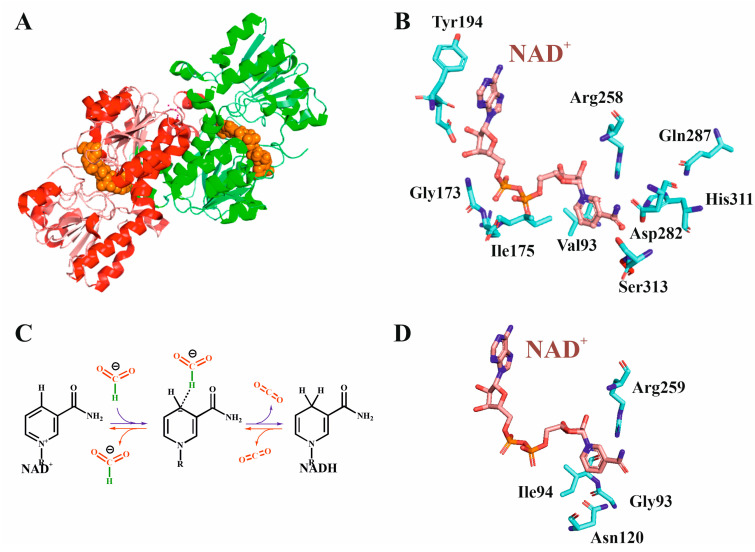
(**A**) Three-dimensional structure of *Cb*FDH with NAD^+^ and azide anion (6D4C pdb code [90]). The two dimers are colored in red and green, while the NAD^+^ cofactor is colored in orange. (**B**) Active center of *Cb*FDH showing the arrangement of the cofactor (pink) as well as the most relevant amino acids closest the NAD^+^, as discussed in the text. (**C**) Mechanism of action of NAD^+^-dependent FDHs for formate oxidation (blue arrows) or formate reduction (inverse sense, red arrows); the NAD nicotinamide group in its oxidized and reduced state is displayed. (**D**) Active center of *Ct*FDH (6T8Z pdb code [91]) showing the four residues mutated in both works cited in the text [92,93], the numbering corresponding to *Ct*FDH.

Metal-independent FDHs belong to the family of D-specific 2-oxoacyd dehydrogenases. They are present in bacteria, yeast, plants, and mammals, are globular proteins with ca. 350–400 amino acids, depending on the species, and usually form homodimers [25,26,86]. Each FDH monomer contains two domains, one destined to allocate the substrate (the catalytic domain) and another pocket that allows for the binding of the NADH cofactor. The function of metal-independent FDHs is generally associated with mechanisms for obtaining energy from the oxidation of formate anions in methanogenic pathways (backward reaction 5), that is, these FDHs are machines efficient in catalyzing backward reaction, although much less effective in performing CO_2_ reduction [25,26,84,87]. The cofactor of all known metal-independent FDHs is NAD(P)^+^/NAD(P)H, and, consequently, they are also called NAD(P)-dependent FDHs. FDH from the methylotropic yeast *Candida boidinii* (*Cb*) is the most studied and best characterized nondependent FDH, since it was the first FDH expressed in *Escherichia coli*, it is commercially available and relatively inexpensive [88,89]. As shown in Figure 5A, the enzyme (364 amino acids) consists of 15 α-helices and 13 β-strands [90]. There is a deep groove between both domains that allows both the substrate and the cofactor to be bound in this cavity with short contacts between them. Figure 5B displays the active site of the enzyme, including both the NAD^+^ binding site and the most significant amino acids concerning catalytic properties. NAD^+^ cofactor strongly binds the protein through its adenine, ribose, and phosphate moieties in such a way that it can only be removed by extensive washing with, for instance, 0.2 M sodium chloride. Formate binds closely to the nicotinamide group through the positively charged residue Arg258. The mechanism of formate oxidation (Figure 5C) consists of hydride transfer to the nicotinamide oxidized group and the release of the CO_2_ formed. The intermediated anion is stabilized by an arginine (Arg258 *Cb*FDH numbering). The groove where the nicotinamide group and the formate anion are located is hydrophobic; hence, the hydride anion cannot interact with the solvent, so the reaction can take place. Both high formate and NAD^+^ strong binding with positive residues of the enzyme (Gln287, His311) reduce the ability to exchange the products of the reaction. In turn, this is one of the main factors that decrease the efficient recycling of the cofactor, making these FDHs, in general, not excellent biocatalysts for CO_2_ reduction.

With wild-type *Cb*FDH being not ideal for forward reaction 5 [94,95], specific mutants increase its capability to capture CO_2_. For instance, the double mutant V120S-N187D was shown to substantially increase CO_2_ reduction [96]. Other FDHs have *k*_cat_ and *k*_M_ values that are higher than wild-type *Cb*FDHs and its mutants (Table 2). Choe et al. studied *Thiobacillus* sp. KNK65MA FDH (*Ts*FDH) and concluded that it presented 84-fold higher catalytic efficiency for CO_2_ reduction than *Cb*FDH [67]. NAD-FDH mutants with higher efficiency have been successfully designed [89,96]. Binay and coworkers obtained and purified mutants of *Candida methylica* (*Cm*FDH) and another four mutants from *Chaetamium thermophilum* (*Ct*FDH) in amino acid positions close to the cofactor binding [92,93]. The highest activity was found for the Asn120Cys mutant in *Ct*FDH, for which the *k_cat_* value increased 6.5-fold, the same increment observed for the *k*_M_ value, which indicated that the efficiency of CO_2_ reduction was due to the lower affinity for the substrate, that is, for the ability to release formate. As observed in Figure 5D, Asn120 is located close to the nicotinamide NAD group; specifically, the amide nitrogen of Asn120 is as close as 3.8 Å from the nicotinamide ring. Its mutation by a smaller cysteine residue introduces more space in the active site and, consequently, higher conformational flexibility, which facilitates the release of formate anion [92]. His96 interacts with the hydrogen carbonate anion stabilizing the hydride in the transition state, as confirmed by molecular dynamics performed on the double mutant. Based on kinetic and molecular dynamic studies, the authors concluded that subtle structural changes around the Asn120 position allowed for the location of two molecules of hydrogen carbonate instead of one of formate, favoring the CO_2_ forward reaction taking place. On the other hand, replacing key residues G93H/I94Y in *Cm*FDH, located in the catalytic pocket of FDH, increased the catalytic efficiency (*k*_cat_/*k*_M_) of the wild-type protein 5.4-fold for the reduction of HCO_3_^−^. Here, *K*_M_ values do not vary significantly, while *k*_cat_/*k*_M_ does. The authors suggested that there is a reorganization in the active site that enlarges the space and allows for the reactant (carbonate anion) to adopt a better orientation for catalysis, and so it becomes easier for the HCO_3_^−^ to reach the nicotinamide ring and the reaction is produced in a faster way [93].

### 3.2. Metal-Dependent FDHs

FDHs containing metals constitute the other large set of FDHs [28,84,116,117]. All metal-dependent FDHs catalyze the interconversion between formate and CO_2_. The sense of the reaction (Equation (5), Scheme 1) depends on the external conditions. In general, in biological conditions, formate oxidation (backward reaction 5) is favored, and thus, some organisms obtain their energy from this exergonic reaction. However, metal-dependent FDHs can also catalyze CO_2_ reduction, and most of them do so, although to a different extent. There are a small number of microorganisms (hydrogenotrophic methanogens, *Euryarcheota*, and autotrophic acetogens, bacteria) that use FDH not for generating energy (i.e., not for oxidizing formate), but for using C_1_ carbon species as a primary source of their carbon metabolism [116,117]. All these microorganisms have metal-dependent FDHs.

**Figure 6:**
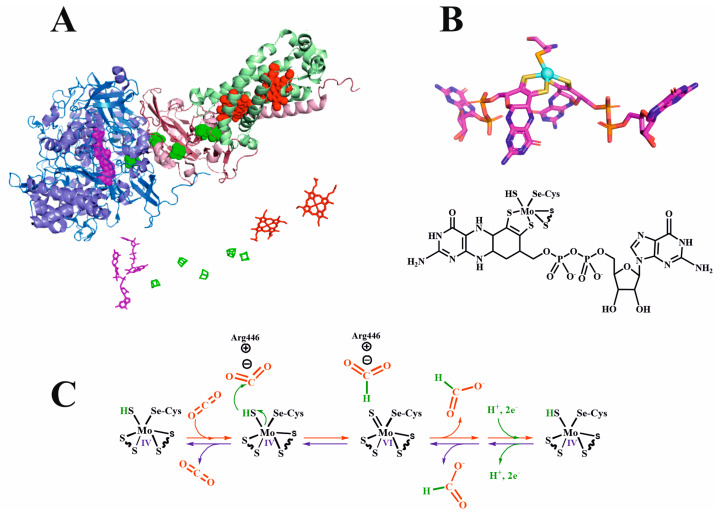
(**A**) **Up**: three-dimensional structure of FDH N from *E. coli* (1KQF pdb code [119]). The three polypeptide chains are shown in blue (chain A), pink (chain B), and green (chain C); the molybdopterin (in chain A), the five F_4_S_4_ clusters (in chains A and B), and the two heme (chain C) metal cofactors are displayed in pink, green, and red, respectively. (**A**) **Down**: a detailed picture of the cofactors at the same scale. (**B**) **Up**: graphic of the molybdenum cofactor in FDH N from *E. coli*. (**B**) **Down**: chemical formula of the Mo cofactor. (**C**) FDH mechanism of action for CO_2_ reduction (red arrows) or formate oxidation (inverse sense, blue arrows) [27]; the direction of the reaction depends on the experimental conditions (see text for details).

This type of FDH is much more complex than NAD-FDHs. Indeed, they have more than 700 amino acids arranged in different domains that, in turn, contain several cofactors and/or metal centers such as ferredoxins, heme groups, flavin mononucleotides, etc., depending on the species [118]. For instance, FDH N from *E. coli* comprises three domains (Figure 6A): the α-domain, which contains the Mo cofactor (see below) and one [4Fe-4S] cluster; the β-domain with four [4Fe-4S] centers; and the γ-domain, with two *b*-hemes (Figure 6B) [119]. These enzymes receive the electrons from these metal clusters and not necessarily from NADH (although, in some cases, NADH can also be the cofactor), and thus, these FDHs are called nondependent NADHs. Excellent reviews describing the three-dimensional structures of these FDHs, their metal centers, their functions, and the biotechnological achievements of these enzymes have been published [28,84,116,117].

Importantly, despite their heterogeneity, these FDHs share common features concerning the active center. They all contain a molybdenum or a tungsten metal ion bound to two dithiolene atoms provided by two pyranopterin guanidine dinucleotides, a sulfur or selenium donor atom, and a disulfide anion (Figure 6B) [104]. CysSe residue is present in both Mo- and W-FDHs; hence, it is not specific to a determined metal. On the other hand, according to kinetics parameters (*k*_cat_ and *k*_M_) the presence of CysSe instead of the native amino acid cysteine, is not crucial for FDH activity.

Metal-dependent FDHs catalyze Equation (5) (Scheme 1) in both directions; however, unlike wild-type NAD-dependent FDHs, they are much more efficient in catalyzing forward Equation (5) than the latter, clearly evident in Table 2. This table presents the kinetic parameters, as well as the composition of the active site and cofactors, specifically for metal-dependent FDHs. Hence, for biotechnological applications, that is, for reducing CO_2_, metal-dependent FDHs are much more active and, hence, more attractive than non-metal FDHs.

What are the key factors that enable metal-dependent FDHs to reduce CO_2_ efficiently? These issues have been extensively studied in the literature [26,27,28,29,84]. Here, some notes on the crucial aspects are commented upon. First, the existence of different redox centers acts as a corridor for efficient electron transfer toward the CO_2_ molecule. Second, and importantly, there is presence of a sulfido group that accepts a hydride anion (see Figure 6C). It is well known that, in Mo/W enzymes, a sulfido group accepts a hydride. In FDHs, metal oxidation states change from IV to VI; when they are in a reduced state, ligands tend to be protonated, while tending to deprotonate in the Mo/W(VI) oxidation state. Thus, metal sulfido can act as a donor/acceptor hydride. Indeed, spectroscopic studies are consistent with the transfer of a hydride from a sulfur atom [27,84,116,118]. On the other hand, there is no evidence of the direct coordination either of CO_2_ or formate directly to the metal center. Altogether, this facilitates the acceptance/donation of a hydride directly towards the CO_2_ carbon, which has an electronic net deficiency, and so is prone to attack by anions. It is also remarkable that the tungsten or molybdenum metal ion are indistinguishable concerning the catalytic activities while the presence of selenium cysteine does not appear to be relevant in CO_2_ reduction.

Several schemes of the reaction have been proposed for the mechanism of action of these FDHs [120,121]. However, it is robustly supported that CO_2_, rather than HCO_3_^−^, is the substrate of FDHs for forward Equation (5) [104,122]. This reaction takes place by abstracting (or adding, reverse reaction) a hydride anion, without the intervention of any oxygen atom [123]. Concordantly, the mechanism of action should consider these two fundamental facts. Moura et al. proposed a mechanism for the forward reaction in which the reduced CysSH coordinated to the Mo ion attacks the carbonyl atom and hydride transfer takes place (Figure 6C) [27]. The formate anion is then stabilized by the positive charge of Arg446 (FDH N from *E. coli* numeration), and afterward, when the protein is again reduced by the other cofactors and by the addition of another hydride to the coordinated Cys, the formate is released. In contrast, the reverse reaction is also produced by the opposite hydride attack from the formate anion on the same Cys (in this case, oxidized).

## 4. Improving CA Performance: Enzyme Immobilization

For industrial and biotechnological applications to be profitable, enzymes must be as stable and reusable as possible. CA and FDH in solution, like all soluble proteins, behave as solutes with full mobility in the solvent. Although an aqueous medium is, in general, the most suitable for enzyme action, the stability and activity of enzymes in this medium usually decrease rapidly. In addition, the use of enzymes in solution is always constrained by strict pH and temperature conditions. More importantly, enzymes in aqueous solutions can only be applied in the cycle of a specific reaction; hence, their applicability at the industrial level is highly limited. In contrast, enzymes immobilized on solid or gel supports extraordinarily increase their stability, amplifying the range of action of the biocatalyst conditions and the possibility of using more drastic, usually more efficient, reaction conditions (for instance, increasing temperature) [124,125,126]. This immobilization allows for their easy separation from reactants and products, and, consequently, enzymes in this form can be reused for posterior cycles [127,128]. This drastically reduces the cost of the whole process, regardless of the biotechnological industrial reaction. Both CA and FDH have been immobilized on different supports. Based on the immobilization method, the following approaches can be considered: physical adsorption, covalent binding, entrapment, encapsulation, and crosslinking.

**Figure 7:**
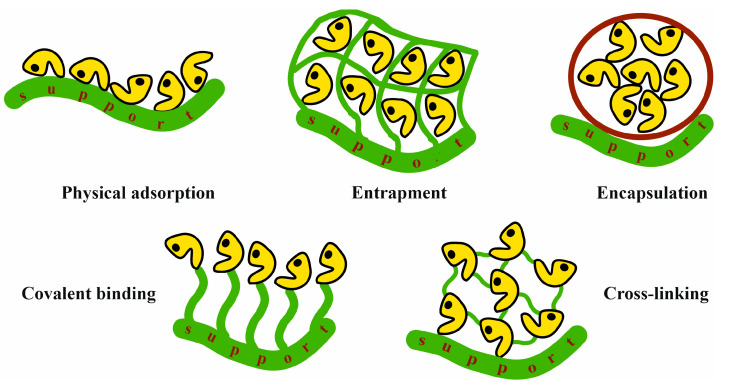
Different methods of enzyme immobilization.

Excellent reviews on CA and FDH immobilization have recently been published [129,130,131,132,133,134,135,136,137,138,139]. Here, some illustrative cases regarding the relevance of immobilization in enzyme stabilization are highlighted according to the immobilization method (Figure 7). Because all the examples provided in Section 6 are related to CO_2_ reduction by immobilized FDH, here, the focus is on CA immobilization. Table 3 summarizes relevant studies on CA immobilization using these methods.

### 4.1. Physical Adsorption

Physical adsorption was the first method used to immobilize enzymes [161]. It consists of affixing the protein onto a solid matrix utilizing hydrophobic (van der Waals), electrostatic (ionic), or hydrogen bonding interactions [162]. Physical adsorption can involve partial conformational changes and/or denaturation of the enzyme; thus, special attention must be paid to avoid these events and confirm that the whole activity of the enzyme is retained after immobilization. The types of functional groups on the surface that produce the adhesion of the enzyme to the matrix is one of the crucial aspects of this immobilization method [124]. These groups can contain hydroxyl, carboxyl, amino, sulfhydryl, or imidazole groups, among others, and produce interactions with the rest of the amino acids of the protein. Weak interactions are optimal since strong ones could result in enzyme conformational changes or even denaturation. Indeed, weak interactions are nonspecific and reversible; therefore, proteins can be easily recovered. For instance, if the interactions are electrostatic, free protein can be released into the medium by simply increasing the ionic strength of the solution.

Pore structure can affect enzyme accessibility and, consequently, both the quantity and activity of the immobilized enzyme. Finally, the surface area is also a critical factor: the higher the surface area, the larger number of adsorption sites, increasing both the quantity of immobilized enzyme and the global activity of the carrier. Physical adsorption can increase the stability of the enzyme against changes in pH, temperature, or organic solvents; the enzymes can be easily separated from the reaction mixture, making reuse easy. Moreover, in some cases, adsorption can increase the activity of the enzyme due to stabilization of the active conformation of the enzyme. On the other hand, physical adsorption can sometimes result in loss of enzyme activity if the microenvironment is not adequate or in a decrease in activity due to diffusion limitations of the reactants towards the enzyme active center that can reduce the rate of substrate conversion. Finally, the cost of the enzyme immobilization process can be high when applied, in particular, on an industrial scale.

Mesoporous silica and aluminosilicates are excellent candidates for enzyme immobilization using adsorption methods [163]. Here, the size and structure of the pores are crucial. Mesoporous silica with larger pores can allow for higher enzyme accessibility to the adsorption sites, which typically increases the quantity of the immobilized enzyme and, consequently, its activity. Wanjari et al. immobilized CA in an ordered mesoporous synthesized aluminosilicate, obtaining acceptable kinetic values for the biocatalyst compared to the free enzyme, remaining stable for more than 25 days [140]. Yu et al. immobilized CA in silica functionalized with carboxylate groups, which provided a very high degree of enzyme uptake, and, although the enzyme slightly changes its conformation with respect to the free enzyme, their activities were almost equivalent (95.6% that of the free enzyme versus that of the immobilized enzyme) [141]. Vinoba and coworkers adsorbed bovine carbonic anhydrase (BCA) inside octa(aminophenyl)-silsesquioxane silica nanoparticles modified with silver or gold, which continued to be active after 20 recycling runs [142]. This adsorption approach has been developed extensively, a recent example being the fixation of CA together with FDH to produce formate in silica nanoparticles modified by polydopamine and polyethylamine [143]. Here, the production of formate was expedited up to 30-fold with respect to the free enzyme and activity was retained at 86.7% after 10 cycles.

Colloids are another type of support used to adsorb CA [138]. Crummblis et al. immobilized CA in gold sols, obtaining an enzyme with levels of activity comparable to that of the native one [164]. Curiously, denatured CA was also immobilized in modified Sepharose and subsequent enzyme renaturation using a cycle of heating and cooling, resulting in an active enzyme with elevated activity [144]. CA immobilization via electrostatic adsorption has been studied with nanoparticles using different charges [145]. Positively charged nanoparticles do not adsorb human CA II, while negatively charged ones do, showing kinetic activity that depends on the degree of hydration of both the enzyme and the particle surface. More recently, CA was fixed onto two different types of membrane via layer-by-layer assembly: the first with a porous membrane and the second without [146]. The carbonation rate of the porous membrane was three times higher than that of the enzyme alone. On the contrary, the nonporous membrane was less active (70–90%) than the native non-immobilized enzyme. The adhesive properties of the polysaccharide chitosan modified with different compounds have also been employed to immobilize CA [153,165]. Matrixes of chitosan with different coating methods and a given textile package have been shown to adsorb CA in a “drop-in-ready” method, with high efficiency for CO_2_ scrubbing. The physical properties of these matrixes for CO_2_ capture were maintained for more than 31 days, with high efficiency (>80%) at moderate temperatures.

### 4.2. Entrapment and Encapsulation

Immobilization by entrapment occurs when a polymer, gel, or metal organic framework (MOF) is generated in the presence of an enzyme [132,166]. In such cases, the protein can remain trapped within the hollows of the polymer. The nature of these interactions is not chemical in origin, but rather physical. The proteins have free movement at a local level, but the motion is highly restricted to the confined hollows, and most of the molecules are isolated and interact only with the matrix. Drozdov’s group immobilized CA into the pores of different sol–gel magnetite with singular magnetic properties using this method [147]. They studied the physical properties of the new material as well as the overall structure of the enzyme, mainly using infrared spectroscopy, concluding that the protein maintains its 3D arrangement in the generated nanoparticles. The immobilized enzyme was stable and catalytically active at 90 °C, which is the temperature at which the native free enzyme is completely denatured.

Encapsulation is similar to entrapment in the sense that molecules are also free in solution and their movements are restricted; however, molecules are captured in higher bags where they can interact with each other. Sol–gel matrices have also been employed for the encapsulation of CA with excellent results. Polyurethane foam has also been employed to entrap not only the enzyme itself but also *E. coli* cells expressing CA [148]. Indeed, whole-cell catalyst CO_2_ hydration activity was measured by comparing both sole and whole-cell immobilized enzymes with respect to the free enzyme. The efficiency of hydratase activity (Equation (4)) was 16-fold higher for the whole-cell immobilized enzyme than for the free enzyme. Interestingly, the activity of the whole cell trapped in the PUF was approximately 100% for at least nine cycles. MOFs are structurally ordered materials formed from inorganic complexes bridged by organic ligands that are projected in three dimensions [167]. Hollows of defined sizes are arranged monotonously in MOFs. MOFs are employed in a multitude of applications, with the immobilization of proteins being one of the most promising [168,169]. CA has been encapsulated in different MOFs with different features, most of them being zeolites constituted by imidazolates, with acceptable or excellent results [149,154,170]. The enzyme encapsulation within MOFs generates enzyme diffusion through windows that have a smaller size than the cavity. Whether or not the term encapsulation can be properly applied to the immobilization of enzymes in MOFs depends on the ratio between the pore and the enzyme size. In any case, an MOF based on Ni(II) showed a high degree of reusability for CA, retaining more than 65% of its activity after eight cycles [149]. Zinc has also been used as a base for MOFs to immobilize CA. In this case, the Zn-OH groups of the hollow imitate the active site of the enzyme, which permits high CO_2_ capture efficiency [171,172]. MOFs containing several lanthanides have also been employed. In this framework, the existence of a high level of electrostatic interactions substantially increases capacity for CO_2_ uptake [173]. Here, taking advantage of the lanthanide contraction, the specific dimensions of the hollows can be modulated, with the Eu(III) derivative having the highest affinity towards carbon dioxide. In all these examples, infrared spectroscopy is one of the key techniques for characterizing the degree of CO_2_ capture, as well as the distortions of the framework.

Ionic liquids (ILs) have also been employed to immobilize CA, although to a lesser extent [150,174,175,176,177]. While CO_2_ is nonpolar, owing to the difference in electronegativity of the carbon and oxygen atoms, the charges of ILs can absorb CO_2_ to a high degree; thus, this is a field fertile for exploitation. Recently, CA was immobilized in poly(ionic liquids) (PILs) by mixing the monomer hydrophobic IL 1-vinyl-3-hexylimidazolium bis(trifluoromethylsulfonyl)imide with an ethylene glycol derivative that had previously been polymerized using crosslinking [133]. After generating the PIL, CA was entrapped within the hollows of the polymer. The yield of the resulting CA was highly dependent on the size of the porous material and the degree of humidity (the dry PIL was less efficient). The authors also tuned the degree of particle size using previous sonication and studied the kinetic parameters of CA-PIL. These values were comparable to that of the free enzyme, although the entrapped CA was stable for a month without detectable loss of activity, while the free enzyme decreased its activity by more than 30%. The CA-PIL was reused for five cycles with 60% activity.

### 4.3. Covalent Binding and Crosslinking

Covalent binding implies the formation of bonds between the groups of adequately functionalized supports and an enzyme. This is, by far, the most extended approach for immobilizing enzymes, particularly for CA [152,155,178,179]. Several protein functional groups can be used for this purpose. CA has been covalently bound to different supports by its amine groups by reaction with glutaraldehyde [156,180]. Generally, mesoporous supports containing hollows of controlled sizes are grafted with amine groups to obtain solid materials that are prone to covalently binding to enzymes using glutaraldehyde. This was carried out with the support SBA-15, in which three different amine compounds were inserted, followed by covalent immobilization of HCA [142,152,181]. The resulting material was morphologically characterized, and its activity, thermal stability, and reusability were also determined, obtaining better results than those of the free enzyme. Kimmel et al. immobilized CA on the surface of propylene fiber membranes. These membranes, commercially available, were coated with a siloxane layer and functionalized with amine groups. Posteriorly, CA was attached to these fibers via glutaraldehyde crosslinking under two conditions: with and without chitosan tethering. Then, the authors applied these fibers to CO_2_ removal, finding enhancements of 115% and 37% versus the buffer and the blood controls, respectively [157]. Moreover, carboxylic groups activated by carbodiimide and *N*-hydroxysuccinimide agents were used to covalently immobilize CA in microtubes [182]. The resulting immobilized enzyme enhanced its ability to sequester CO_2_ with respect to the free enzyme.

Finally, the crosslinking method is actually a special way of covalent binding. The proteins are bound to other large proteins to form high molecular weight complexes without any solid support or, properly, the enzymes themselves being a solid support. Typically, the protein is precipitated with the appropriate agent and then crosslinked, which can be performed with a purified protein or an extract of a still unpurified enzyme. This method confers high stability and a high degree of enzyme recovery. CA has been immobilized via crosslinking in numerous studies. Recently, Xu et al. encapsulated crosslinked CA in alginate beads and confirmed that this crosslinked CA enhanced the growth of microalgae cultures [183]. The crosslinked CA was stable during 10-cycle assays. Magnetic nanoparticles aggregated with CA were obtained by crosslinking the enzyme with glutaraldehyde, improving the yield of absorbing CO_2_ up to 3.4-fold with respect to the free enzyme and retaining 95% activity after five cycles of reuse [158]. These magnetic nanoparticles are amply used because they allow for very simple separation and recovery of the biocatalyst by using an external magnetic field. They are considered excellent carriers and supporting matrices for enzyme immobilization, providing several advantages for the design of biocatalytic processes (i.e., large surface area, large surface-to-volume ratio, high mass transference, etc.). More recently, Chang et al. crosslinked CA and geopolymer microspheres with glutaraldehyde and performed a detailed study on the morphology, stability, and activity of the immobilized support [159]. After 60 days of storage at 25 °C, immobilized CA still presented 28.9% activity, whereas free enzyme activity was less than 10%. CA and FDH have also been crosslinked to reduce CO_2_. Zhang et al. used microbial transglutaminase (MTG) as the crosslinking medium for CA and FDH labeled with peptide tags and previously expressed in *E. coli* [160]. MTG catalyzes the formation of an isopeptide bond between the ε-amino group of lysine and a glutamine. The authors studied the activity and reusability of several crosslinked particles with different tags at CA/FDH ratios of 1:1, 1:2, and 1:3. Because CA is much more active than FDH, it is expected that the lower the CA/FDH ratio, the higher the formate yield obtained. However, the optimal found CA/FDH ratio was 1:2. The authors attributed the lower yields obtained for a 1:3 ratio to FDH steric hindrances in the crosslinked aggregates.

## 5. Carbon Capture Storage and Utilization: State-of-the-Art, Costs, and Perspectives

Methods for CO_2_ capture are classified into precombustion, postcombustion, and oxy-combustion processes [12,184]. The precombustion approach is related to hydrogen gas production. This is obtained in numerous industrial processes such as electric power generation, ammonia or fertilizer synthesis, and petroleum refinement. The precombustion process refers to the conversion of the primary solid fuel (coal or biomass) by reforming it into a mixture of CO and H_2_ gas (syngas). This gas reacts with the water stream at high temperatures and pressures to produce CO_2_ and more H_2_ (water–gas shift reaction). Finally, CO_2_ is captured using several methods. Postcombustion CCS methodology denotes all the processes employed to capture CO_2_ from exhaust gas (its major component being nitrogen) resulting from industrial chemical processes. CO_2_ gas is emitted at relatively low temperature and pressure. Oxy-fuel combustion consists of the oxidation of fuel using pure oxygen instead of air, obtaining an almost pure CO_2_ atmosphere without nitrogen gas. Most CCS methods have been developed for postcombustion gases and are referred to here, except where otherwise indicated. In many cases, the methodology can be the same for both post- and precombustion approaches, although with different designs depending on the P/T conditions of the exhaust gases. Physical adsorption and absorption [185] (geological storage [186] probably being the most relevant among absorption approaches) and cryogenic distillation [187] are the main methods used for CO_2_ CCS.

Global CO_2_ emissions from combustion processes grew by 0.9% in 2022, reaching a total of 36.8 Gt [188]. Energy used in industry, agriculture or land use, buildings, transport, direct industrial processes, waste, and others with 37.8%, 18.4%, 17.5%, 16.2%, 5.2%, 3.2%, and 1.7%, respectively, are the contributions to CO_2_ emissions by the different sectors [189]. Only ca. 40 million Tm, i.e., 0.1%, was removed from the atmosphere using CCS methods in 2019. Thus, we are still very far from being efficient in eliminating the CO_2_ expulsed into the atmosphere.

The estimated present costs of CO_2_ Tm removal vary nowadays from 40 to 80 USD depending on the method [190]; however, the net contribution to CO_2_ elimination from the atmosphere is difficult to calculate, since net contributions in the whole process have to be taken into account. Hepburn and coworkers analyzed the perspectives, including cost, for different methods of CO_2_ utilization [190]. They analyzed ten different methods of CO_2_ utilization. For instance, chemical production, particularly the generation of urea, on one hand, and the production of polycarbonate polyols, on the other, are two fields in which CO_2_ capture can be exploited. They estimated that CO_2_ utilization in chemicals in 2050 could be around 0.3–0.6 Gt CO_2_/yr with costs ranging from −80 to 320 USD per Tm of CO_2_ (a negative value would indicate an additional economic benefit, while a positive value indicates that the cost of capturing and utilizing CO_2_ would be higher than the value generated from it). Fuels, that is, CO_2_-methanol plants, were also considered in their study, although they stated that many different scenarios can vary their prospects from 1 to 4.2 Gt/yr in 2050 and, in terms of cost, from 0 to 670 USD per Tm of CO_2_.

## 6. Carbonic Anhydrase in Carbon Capture Storage

Although CA is not used in all previous technologies, its use is extended or has a good perspective in others, mainly chemical adsorption and mineralization, whose description is commented on below. Table 4 describes relevant studies performed with CA in CCS research.

### 6.1. Chemical Absorption

Chemical absorption has been the most used CCS method for decades [203]. This procedure involves the scrubbing of exhaust gas at low pressures and temperatures with alkaline solutions typically containing amines and/or carbonates or hydroxide solutions [203]. Amines are weak bases that can capture protons from Brönsted acid CO_2_. The reactions of primary/secondary or tertiary amines produce carbamates or bicarbonate anions, respectively, according to the reactions described below.

The amines typically used for these purposes are alkanolamines. An alcohol group increases water solubility and decreases vapor pressure compared to analogous amines. The main chemical solvent used as an absorber is monoethanolamine (MEA). The solutions typically consist of an aqueous solution of 20–30 wt% MEA. CO_2_ is captured at low pressure (ca. 1 bar) and in a mixed gas containing other gases, such as N_2_, SO_x_, and NO_x_. Secondary amine diethanolamine (DEA) and tertiary amine *N*-methyl diethanolamine (MDEA) are also amply used amines.

Several points should be considered when choosing a suitable solvent for CO_2_ sequestration. First, the enthalpy of the reaction: the higher the enthalpy of the reaction, the higher the cost of solvent regeneration. This is a crucial point because it is estimated that 60–80% of the costs of these processes arise from solvent regeneration [204]. The enthalpy of (exothermic) reactions 6 and 7 increases from tertiary to secondary and primary amines; thus, primary amines improve both energetic and economic costs. Moreover, the power of corrosion also follows the same order (primary amines are the most corrosive). Another decisive issue is their ability to load CO_2_. Tertiary amines have the highest capabilities in this regard [205]. According to these thermodynamic aspects, tertiary amines are the best ones to use. However, another crucial point is the kinetics of CO_2_ sequestration; indeed, tertiary amines have low reaction rates and are kinetically much more inert than primary amines [206,207]. Owing to the slow kinetics of tertiary amines, primary amines (or secondary amines) are currently preferred. However, the high costs of cooperation and maintenance due to the ease of amine degradation and the formation of highly corrosive salts are drawbacks when operating with amines.

Consequently, for kinetic reasons, primary amines, specifically MEA, are by far the most widely used alkanolamines in the industry. Numerous plants have been developed using MEA solutions. These systems are typically coupled with industrial processes and CO_2_ is captured with postcombustion gases. For instance, their use is extended to the iron and steel industries (responsible for approximately 31% of all industrial CO_2_ emissions). These plants can recover 85–95% of the CO_2_ in gas. As an example, a steel production plant recently established by Emirate Steel Industries has a yield plant using CCS based on amine absorption that captures 0.8 Mt CO_2_/year [208].

In the last decade, CA has been revealed as a tool for accelerating CO_2_ uptake in chemical absorption processes. Gundersen et al. studied CA stability and activity for a long time (150 days) as a function of pH, temperature, and the solvent, combining MEA and MDEA solutions, among others [191]. They concluded that CA was suitable for these purposes; the biocatalyst was stable and active between pH 7 and 11, with maximum activity at 40 °C. In addition, the enzyme preserved its activity between 12 and 91% of the original activity depending on the solvent employed. The absorption of CA was also accomplished in MOF ZIF-L-1 in the presence of MDEA [198]. In ZIF-L (a zeolitic imidazolate framework), the imidazolate groups enhance CA immobilization, and CO_2_ uptake is hence greatly increased. The authors highlighted that this new MOF obtained excellent CO_2_ absorption rates at 40 °C and a CO_2_ partial pressure of 15 kPa, while the activity was maintained for six reuse cycles [192]. Additionally, a pilot-scale plant was set up with CA in solution in the presence of MDEA. The authors observed an enhancement in CO_2_ capture in the presence of the enzyme and demonstrated the possibility of translating the laboratory results to higher scales [193]. However, because the free enzyme is damaged by amines, immobilization is necessary. Kim et al. also studied the effect of CA on CO_2_ absorption rates in the presence of MEA and MDEA, although they used a membrane contactor with hydrophobic and hydrophilic supports [194]. This system allows an expanded contact surface to enhance CO_2_ absorption.

### 6.2. Chemical Carbonation

Chemical carbonation is probably the most efficient method for capturing CO_2_. This is performed when CO_2_ is bubbled through an alkaline solution, typically consisting of dissolved KOH or Ca(OH)_2_, where potassium or calcium carbonates precipitate. The limiting step for capturing CO_2_ in postcombustion processes is Equation (3). However, due to Equation (4), CO_2_ capture is much faster in alkaline media since carbonates are formed, and so hydrogen carbonate concentration decreases, and Equation (3) is shifted towards the consumption of CO_2_. Indeed, once bicarbonate anion (soluble) is formed, reactions such as Equations (6) and (7) (amine formation, Scheme 2) occur much faster in alkaline media. Even so, the limiting step, for kinetic reasons, continues to be the CO_2_ gas uptake, as commented previously. Thus, the main challenge in applying alkaline solutions, either amines or carbonates, to the CCS approach is speeding up CO_2_ conversion to bicarbonate [195]. Consequently, numerous studies on CA to increase the mass transfer of CO_2_ capture have been proven not only at the laboratory level, and its feasibility has been demonstrated on an industrial scale [15,205]. Novozymes NS81239 CA (NCA) at 2 μM increased the absorption rate of CO_2_ into potassium carbonate by ca. 30%, augmenting this uptake at temperatures in the range of 40–60 °C [195,196]. Power et al. demonstrated that bovine CA accelerated the carbonation rate of brucite Mg(OH)_2_ from CO_2_ gas by up to 240% [209]. In these studies, CA was supplied as a free enzyme; therefore, its regeneration was not studied. Biological tools have also been used to enhance carbonation. Jin et al. accelerated calcium carbonate precipitation by employing *Bacillus mucilaginosus* on steel slag powder [197], increasing the carbonation degree from 66.34 to 86.25%. Moreover, the mechanical properties and durability of the treated steel slag were enhanced. The CA immobilization, as described in the previous section, strongly improves the reusability of the enzyme as well as the chemical carbonation. For instance, Jo and coworkers proved the suitability of CA encapsulated in a biosilica matrix, obtaining good yields for carbonation compared to the free enzyme [198].

(6) $${CO}_{2}\left(aq\right)+{2R}_{1}R_{2}NH\;⇄R_{1}R_{2}COO^{-}\left(aq\right)+R_{1}R_{2}NH_{2}^{+}\left(aq\right)$$

(7) $${CO}_{2}\left(aq\right)+R_{1}R_{2}NR_{3}\;⇄HCO_{3}^{-}\left(aq\right)+R_{1}R_{2}R_{3}NH_{}^{+}\left(aq\right)$$

### 6.3. Mineralization

Biomineralization is a very slow and exothermic process by which carbonate minerals are formed from silicates and CO_2_ under basic conditions [210]. The starting silicates usually contain divalent metals such as Ca(II) and Mg(II) or trivalent metals such as Fe(III). This event occurs in nature on a regular basis and is responsible for the formation of inorganic structures in living organisms such as exoskeletons in protozoa, algae or invertebrates, and shells or plant mineral structures. It is also responsible for the presence of large amounts of limestone on the Earth’s surface [211,212]. When trying to emulate biomineralization, which takes place over very large timescales, the main drawback is speeding up the process. Artificial mineralization mimics nature, although in short periods. It involves the injection of CO_2_ directly into geological formations to promote a carbonate-forming reaction with alkaline minerals [213]. This mineral sequestration would be a viable alternative for subsequent storage because the carbonate products formed would not require monitoring owing to their high stability and safety. On the other hand, in-ground or ex situ mineralization is based on the exposure of crushed rock material in a processing plant where CO_2_ is introduced, facilitating the formation of carbonate minerals. Natural minerals or alkaline solid waste can be used [213,214]. The use of natural silicates requires a large amount of material, which implies a very large operational size and an unfeasible economic mineral impact. Ex situ mineralization can also be carried out using alkaline wastes containing divalent metals such as ash originating from the coal or metallurgical industry, cement and concrete wastes, or iron and steel slag [215,216]. This method would reduce not only environmental CO_2_ but also the accumulation of waste from industrial activities, although a major disadvantage in that its capacity is much smaller than that of CO_2_ mineralization from silicates. At laboratory scale, this mineralization has been satisfactorily performed by directly extracting CO_2_ from the air, and its direct extraction by passing the air through cooling towers using NaOH solutions has also been proposed for larger scales [217]. However, the same authors pointed out the elevated costs of this approach on a large scale.

The efficiency of the biomineralization process can also be accelerated by modifying certain parameters such as increasing the temperature, pressure, or retention time. The biomineralization process is also favored by the presence of purines, NaCl, or CA. The presence of CA accelerates the rate of hydration of CO_2_ dissolved in water; therefore, possible modifications to CA to support high pH and temperature conditions without losing its advantageous functionality have been studied [218,219]. On the other hand, some varieties of carbonic anhydrase are inhibited in the presence of high concentrations of hydrogen carbonate, which becomes a problem for its use in industry. However, this can be circumvented, at least partially, by increasing the pH to values equal to or higher than 9.0, conditions under which some CAs are still stable and functional. Immobilization improves the stability of CA at high temperatures or alkaline conditions, as confirmed by Arias et al. when forming calcite in vitro by mineralization using CA immobilized in eggshell membranes [219]. Recombinant CAs have also been used to accelerate mineralization under extreme conditions. For instance, CA from the alkalistable *Aeribacillus pallidus* was genetically modified, achieving acceptable yields in the presence of pollutants such as NO_x_ and SO_x_ [200]. Similarly, CA from the thermophilic bacterium *Sulfurihydrogenibium azorense* was modified, and its half-life was found to be 8 days when the biomineralization process was carried out at 70 °C and 53 days at a reaction temperature of 50 °C [201]. Di Lorenzo et al. studied the effect of CA and a Zr-based MOF in the carbonation process of wollastonite (CaSiO_3_) to produce calcite (CaCO_3_) [202]. Although CA accelerated CO_2_ uptake by the silicate, the total gas absorber quantity was lower than that of the MOF. Jin et al. also took advantage of CA to accelerate the carbonation of γ-dicalcium silicate, which is also present in steel slag. They used a powder containing alkali-resistant CA bacteria, increasing the yield by 19.0% [220].

## 7. Biotechnological Aspects of CO_2_ Reduction

### 7.1. Hindrances to Biochemically Reducing CO_2_

**Figure 8:**
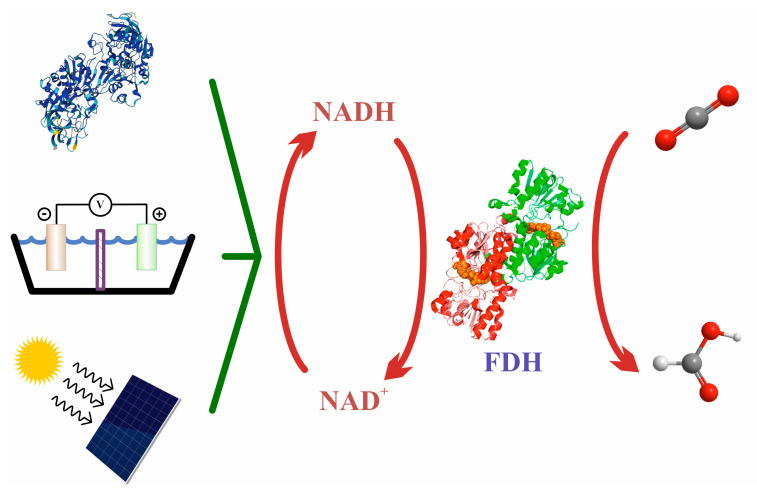
The three different (enzymatic, electrochemical, and photochemical) approaches to regenerating cofactor NADH and producing formate with FDH.

Forward Equation (5) presents several drawbacks that hinder its application outside the natural living environment, that is, employing it with biotechnological aims. First, owing to the low redox potential of the CO_2_/HCO_2_^−^ pair (−430 mV), the reaction is highly endergonic under physiological conditions. Three main strategies have been developed to overcome this problem (Figure 8): coupling thermodynamically favorable reactions in the presence of an excess concentration of the reducing agent [136,221,222,223,224]; coupling the reaction to an electrochemical device that provides electrons from a battery anode (i.e., by supplying electric energy) [225,226,227,228]; and using photosensitive molecules able to absorb light and regenerate redox partners [31,229,230,231,232,233].

Regeneration of the cofactor NADH is crucial for obtaining formate with acceptable yields [234,235,236,237]. As previously mentioned, metal-dependent FDHs do not necessarily accept electrons from NADH (see Figure 6); nevertheless, whatever the cofactor is, either in nature or in biotechnological approaches, it has to be regenerated for adequate progress of the reaction. NADH, the primary source of electrons, is an expensive reactant, and thus economic aspects are also relevant in cofactor regeneration. The approaches to regenerating NADH are similar to those followed to facilitate the thermodynamics of the reaction. Two additional issues related to the oxidation of this cofactor also have to be kept in mind: NAD^+^ can strongly interact through its negative phosphate charges, with positive charges on the active site of the biocatalyst, inhibiting the biocatalyst, particularly in nonmetal-dependent FDHs [238]. Moreover, it is well known that NAD^+^ trends to form dimers (see below), which makes cofactor regeneration impossible [221,234]. Carbon dioxide uptake into the reaction medium is another crucial factor. As commented throughout this text, since CO_2_ is a nonpolar gas, its solubility in water or similar solvents is low and, more importantly, the kinetics of solubilization and mass transfer are very slow [204]. Hence, the employment of systems (specific solvents or solutions) that can incorporate CO_2_ into their structures is decisive. As discussed above (see Section 6.1 and Section 6.2), amines have been used extensively [204].

Ionic liquids, solvents in which positive cations can interact with the oxygen atoms of a CO_2_ molecule through their sole pair of electrons, are also excellent media to solve, in high quantities, carbon dioxide gas [214,239,240]. The selection of the appropriate conditions for the reaction is also crucial. Dealing with a gas, high pressures and low temperatures are ideal conditions for solubilizing CO_2_, while with respect to pH, carbon dioxide solubilization is favored at high pH values (Equations (3) and (4)). However, optimal conditions are determined by FDH stability and activity, and temperatures must thus be moderated (lower than 50 °C), pressures cannot be high, and pH values must be between 6.0 and 7.5, the range at which FDH shows its highest yields. Finally, the use of the adequate enzyme, that is, the FDH of the appropriate organism, is also a key factor in the success of the reaction.

Approaches to circumventing these problems and achieving significant formate yields as a starting point for obtaining value-added chemicals are discussed in the following sections.

### 7.2. Coupled reactions: Enzymatic Multicascades

Equation (5) is reversible although under physiological conditions is highly shifted towards the backward reaction. The first successful conversion of CO_2_ from formate using FDH in a laboratory system was reported by Hopner and collaborators [100]. They used the metal-dependent FDH from *Pseudomonas oxalaticus* as biocatalyst and NADH concentrations in such a way that reaction 5, still being thermodynamically unfavorable, was not completely shifted towards the oxidation of formate. They devised a sealed system containing ^14^CO_2_ and measured the radioactivity of the H^14^COOH formed. In this pioneering study, the kinetic parameters (*k*_cat_/*K*_M_) of the enzyme and its pH activity profile were determined. However, the cofactor was not regenerated and the rates between the forward and reverse reactions in their working conditions were 1:30, with a turnover number for CO_2_ reduction as low as 3 s^−1^, which makes these results insufficient.

**Figure 9:**
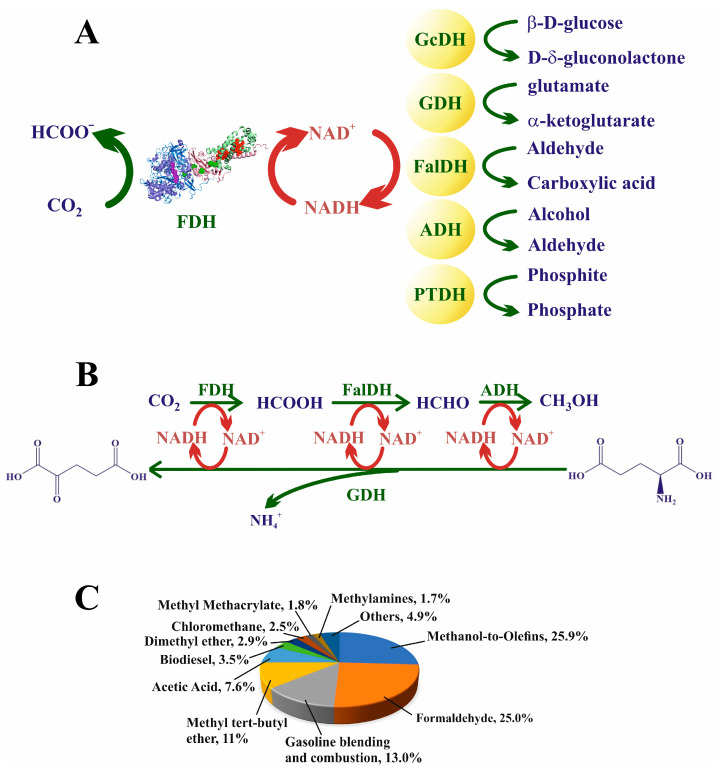
(**A**) Several enzymes whose oxidation reactions have been coupled to FDH CO_2_ reduction for regenerating NADH. (**B**) A classical enzyme multicascade for the reduction of CO_2_ to methanol; here, GDH is coupled for regenerating NADH. (**C**) Uses of methanol at the global level in the year 2020 shown in percentages according to their production and demand [251].

The simplest and most straightforward way to increase formic acid generation is to eliminate the products from the reaction media, that is, regenerating the NADH cofactor using a reducing chemical agent present in the medium. This approach was developed in the 1980s with redox biocatalytic systems [241,242]. The reaction desired to take place is coupled with another “inverse” reaction, in which the reactant is added at high concentrations. Thus, the reaction is thermodynamically favored, and the cofactor is regenerated (Figure 9A). Chenault and Whitsides, pioneers in using this technique with formate dehydrogenase, employed *Cb*FDH to regenerate NADH by coupling formate oxidation with the reduction in lactate to pyruvate using D-lactate dehydrogenase and obtained acceptable results, with nicotinamide residual activity of 55% after each run [242]. However, in their reaction, FDH was used for formate oxidation (backward Equation (5)), and the coupled reaction was used to regenerate NAD^+^. Since then, the reduction of CO_2_ (forward Equation (5)) using FDHs in a conjugated oxidation–reduction reaction system for regenerating the NADH cofactor has been carried out with several enzymes. Yu et al. cloned the FDH gene from *Cupriavidus necator* in *E. coli* and coupled the reduction of CO_2_ with the oxidation of D-glucose to D-δ-gluconolactone using glucose dehydrogenase (GcDH) to regenerate NADH [243] (Figure 9A). The expressed enzyme, an O_2_-tolerant, Mo-dependent FDH, was able to effectively reduce formic acid comparable to that of the nonrecombinant protein. Glutamate dehydrogenase (GDH) is also used to regenerate NADH. GDH catalyzes the oxidation of glutamate to α-ketoglutarate and ammonia, thereby reducing NAD^+^ to NADH. GDH is highly stable over a wide range of pH values and at temperatures as high as 85 °C, as well as being widely commercially available and inexpensive [236,244].

Nonetheless, the most extensive coupled reaction approach is the well-known and widely employed enzymatic cascade reaction that drives from CO_2_ to formic acid, catalyzed using FDH, from this species to formaldehyde, i.e., formaldehyde dehydrogenase (FalDH), the biocatalyst, and, lately, to reduce this molecule to methanol, by the action of the alcohol dehydrogenase (ADH) (Figure 9B). The oxidases mentioned above are typically used for NADH regeneration.

El-Zahab et al. co-immobilized FDH, FaldDH, and ADH together with GDH into polystyrene particles to reduce CO_2_ to methanol [245] (Figure 9B). The NADH cofactor was also immobilized, although separately. The results obtained with the immobilized enzymes were similar to those obtained for the free enzymes; however, importantly, the yield of the reaction was maintained at over 80% after 11 cycles. Ji et al. coupled the same reaction cascade [246], but in their study, the four redox enzymes were entrapped in hollow nanofibers together with CA to facilitate CO_2_ absorption. The methanol yield was 36.17%, retaining ca. 80% of the productivity after 10 reuses, with an accumulative yield of more than of more than 900% for NADH regeneration. In another study using the same cascade, Ren and collaborators encapsulated the same biocatalysts in MOF ZIF-8 and investigated the effect of polyethyleneimine (PEI) on anchoring the NADH cofactor and, hence, on the yield of the reaction [247]. Compared to the free system, the yield of this reactor system increased 4.6-fold and the activity after eight cycles was retained by 50%.

Ionic liquids are known to solubilize CO_2_ [248,249]. Taking advantage of this, Pinelo’s laboratory immobilized the four mentioned enzymes as well as the cofactor in a series of modified ILs composed of choline (CH) and amino acids (CHGlu, CHPro, CHGLy, and CHHis) [250]. They generated a membrane reactor in which the products were removed in situ and the reaction was displaced towards the desired product. The yield of CHGlu increased up to fivefold compared to that of with the control aqueous system when NADH was regenerated. In another study, immobilization of the four enzymes was performed on superparamagnetic nanoparticles. Here, the yield was low (2.3% of methanol per NADH molecule); however, under CO_2_ pressure (126 psi), the reaction yield increased 64-fold after 30 min of reaction [250].

**Figure 10:**
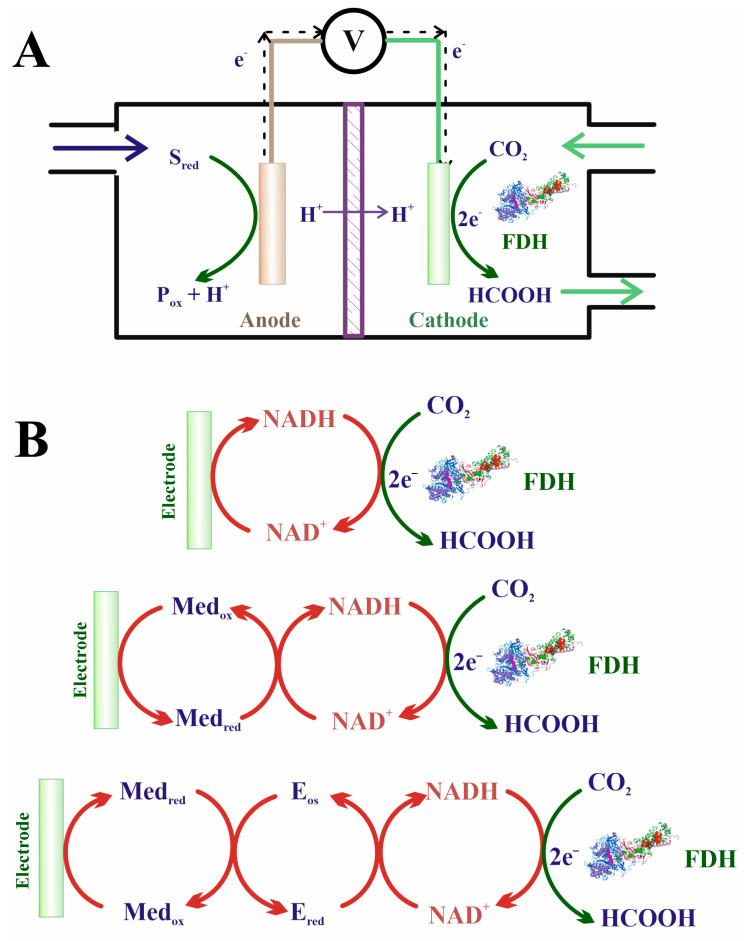
(**A**) Schematic view of an electrochemical device for obtaining formic acid from CO_2_. (**B**) Scheme of three possible mechanisms for transferring electrons from the cathode to the cofactor and finally to FDH: without any mediator species, with a mediator (typically a photosynthesizer molecule), and also with an enzyme intercalated between the mediator and the cofactor.

Phosphite dehydrogenase (PTDH, Figure 10A) has also been used for NADH regeneration in a multicascade approach to obtain methanol from CO_2_. Cazelles et al. studied the effect of regenerating NADH using three different coupled reactions, namely phosphite oxidation, catalyzed using phosphite reductase; glycerol oxidation to dihydroxyacetone, performed using glycerol dehydrogenase; and a natural photosystem (chloroplasts) extracted from spinach leaves that oxidize water to molecular oxygen [252]. They encapsulated the three enzymes in phospholipid–silica nanocapsules and obtained excellent activities with PTDH with respect to the free enzymes in solution (55 times higher activities) under 5 bar of CO_2_ pressure for 3 h, although the other two systems were not so efficient. Singh et al. also employed PTDH for regenerating NADH [253]. They expressed recombinant proteins (FDH, FalDH from different bacteria, and ADH from yeast) in *E. coli* and performed assays with free enzymes in water solution and in the presence of many different cosolvents. IL 1-ethyl-3-methylimidazolium acetate (EMIM-Ac) was found to be the most effective in increasing methanol production. Indeed, the yield was enhanced more than twofold (from 3.28 mM of methanol to 7.86 mM, 6 h of reaction) in the presence of 1% EMIM-Ac because of the ability of EMIM cations to interact with CO_2_, increasing solubility. Finally, lactate dehydrogenase (LDH) was also used to regenerate NADH in a multicascade reaction [254]. This enzyme was immobilized in a sol–gel matrix, and CO_2_ reduction was acceptable after 1 h of reaction, as indicated by the authors.

Using CO_2_ as a substrate for the generation of methanol is an attractive process because of its potential use as a fuel and the multitude of products obtained from it at the industrial level. Figure 9C shows the chemicals produced from methanol at a global level in 2020 [251]. Methanol is a precursor of numerous compounds such as olefines (25.9% of the demand for methanol, the year 2020), formaldehyde (25.0%), gasoline blending (13.1%), biodiesel, and so on. Therefore, it is of great commercial interest due to its potential application both in the energy industry as a fuel and in environmental CO_2_ sequestration to mitigate high atmospheric CO_2_ levels. The methanol production in the year 2023 was 98.9 × 10^6^ Tm and it is projected that by the year 2027, more than 8 × 10^6^ Tm will be obtained from e-methanol (produced from captured carbon dioxide and hydrogen produced from renewable electricity, ca 5 × 10^6^ Tm) and biomethanol (produced from sustainable biomass, ca 3 × 10^6^ Tm). For example, currently, 4000 Tm of methanol is produced annually using biocatalysts based on copper and zinc oxide in Iceland, recycling some 5500 tons of CO_2_ annually. Other approaches to achieving the same effect have also been undertaken in Germany and China [222]. The use of enzymes is an advantage for the conversion of CO_2_ to methanol because of the high selectivity of the catalyzed reaction. This reduction of CO_2_ to methanol is considered a green chemical process and occurs at atmospheric temperature and pressure [253].

### 7.3. Electrochemical Regeneration of NADH Cofactor

NADH can be regenerated at the cathode of an electrolytic cell by applying adequate electric voltage (Figure 10A). This method can be extensively applied and permits the easy separation of products [225,226,227,228]. In principle, as the supported potential difference can be as high as desired within technical constraints, the cofactor regeneration under appropriate conditions could be high, although the complete energy cycle is probably not. If a cell is supported by renewable energy, this approach can also be considered green. In the electrolytic cell, the cofactor can be the primary acceptor of electrons from the electrode (direct electrochemical regeneration, Figure 10B) or, in contrast, other molecules can accept the electrons, the cofactor being reduced by these mediators (indirect mode Figure 10B).

**Figure 11:**
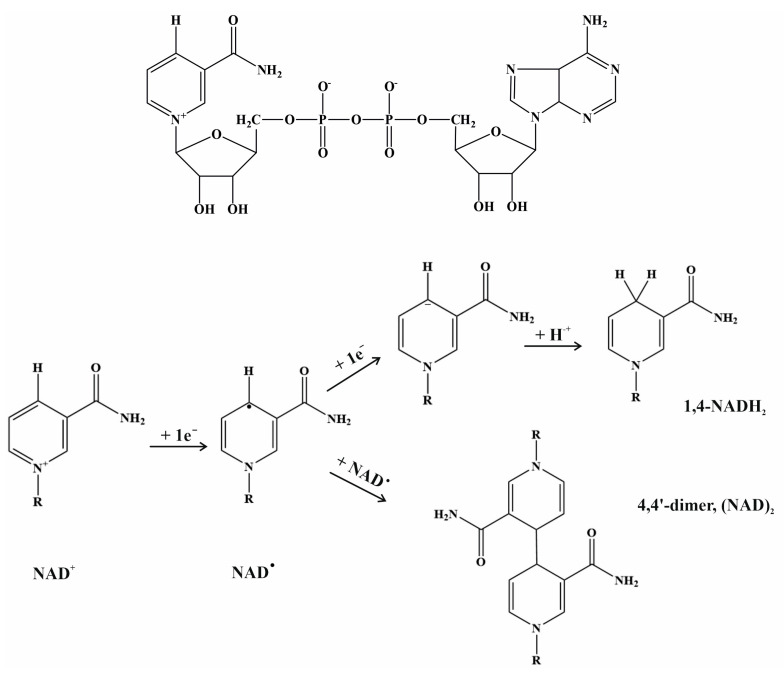
The two reduction steps of NAD^+^. The addition of one electron produces the radical NAD*. This radical can either be reduced by the addition of an additional electron and a proton (up), to produce 1,4-NADH (up) or combine with another radical to generate the 4,4′-dimer (NAD)_2_.

The direct mode has two intrinsic disadvantages that are very difficult to overcome: the formation of (NAD)_2_-inactive dimers and the necessity of using high overpotentials. The electrochemical reduction of NAD^+^ molecule proceeds in two stages (Figure 11). In the first step, NAD^+^ captures an electron and an NAD* radical is formed. In the second step, an additional electron and a proton are accepted by the NAD* radical. However, NAD* can dimerize and, furthermore, due to the adsorption of NAD^+^ onto the electrode, this dimerization process is favored over the uptake of the second electron. The need to use high overpotentials for direct NADH reduction is another limitation of this method. Several studies modifying the electrode nature concluded that mass transfer between the cathode and the cofactor was a crucial step for favoring reduction versus dimer formation. These problems can be partially circumvented by selecting an appropriate electrode [255]. For instance, Ag or Pt electrodes coated on Cu foams were successfully employed for NADH regeneration [256]. The existence of a mediator on the electrode surface is another key point for avoiding dimer formation. Mediators such as (2,2-bipyridyl) (pentamethylcyclopentadienyl)rhodium, [Cp*Rh(bpy)], and methylviologen (MV) have been shown to decrease the overpotential for NADH regeneration, facilitating electron transfer and NADH recovery [221].

In an elegant experiment, Song et al. generated a cysteine residue in FDH from *Thiobacillus* sp. KNK65MA and attached the mutated enzyme to copper nanoparticles (CuNPs) deposited on the electrode surface [257]. Polyethylene glycol (PEG) was then used to crosslink the FDH-NAD^+^ system so that the cofactor could swing from CuNP to the enzyme and vice versa. No mediator was used. This system produced 8.5 mM formate, several times that achieved with a free enzyme.

The enzyme cascade for methanol production from CO_2_ (Figure 10B) has also been widely performed using an electrochemical approach. Addo et al. coupled the multienzyme cascade for producing methanol from CO_2_ to a polyneutral red electrode to regenerate NADH [258]. Electroenzymatic reduction in CO_2_ was also achieved with maximum Faradaic efficiency (99 ± 5%) by the immobilization of Mo-dependent FDH from *E. coli* at the surface of a carbon electrode [259]. Here, reduction was achieved through the mediator cobaltocene being covalently bound to the linear polymer poly(allylamine), which transferred electrons from the cathode to the enzyme. In another interesting study, CO_2_ reduction was electrochemically achieved by using copper deposited in a glassy carbon electrode, the Rh(III) complex [Cp*Rh(bpy)Cl]^+^ (Cp* = pentamethylcyclopentadienyl; bipy = bipyridine) as a mediator and *Cb*FDH as a biocatalyst, with yields threefold higher than those of previous analogous works with copper foil electrodes [260]. Chen et al. also used an Rh(III) complex as a mediator for the electrochemical regeneration of NADH [261]. MOF NU-1006 containing *Cb*FDH was deposited on the electrode surface of fluorine-dopped tin oxide glass. NU-1006 is a mesoporous material with a channel size that can accommodate *Cb*FDH (6 × 4 × 11 nm). The electrode was obtained using a multilayer system in which the entrapped enzyme was the latter and was in contact with the solution containing CO_2_. Regeneration of the cofactor was optimum because of the modified electrode, with a formate generation rate of 79 ± 3.4 mM h^−1^. All these experiments have the advantage of easy regeneration of NADH, due to the possibility of creating a sufficient negative redox potential. However, its translation towards more complex systems, such as a multicascade system coupled to other oxidoreductases, no longer seems biotechnologically accessible.

Barin et al. immobilized *Cb*FDH on polystyrene nanofibers and NADH in a copper foam electrode [262]. Although the activity of this system was inferior to that of the free enzyme, the immobilized enzyme was stable for a long period (41% of the initial yield after 20 days) and had acceptable reusability after eight cycles (53% of the initial activity). The authors also observed an inhibitory effect of NADH at concentrations higher than 0.51 mM. Indeed, several studies have demonstrated the inhibition of FDH by the cofactor at concentrations higher than millimolar. On the other hand, Zhang et al. encapsulated FDH, FaldDH, and ADH in MOF ZIF-8 and used the Rh complex (Cp*Rh(2,2′-bipyridyl-5,5′-dicarboxylic acid)Cl_2_) grafted on the cathode as a mediator to perform NADH regeneration [263]. The concentration of methanol obtained was fivefold (from 0.061 to 0.320 mM) with the encapsulated enzyme compared to the free enzyme. Moreover, when NADH was electrochemically regenerated, an increase of 0.742 mM was observed. This again demonstrated that electrochemical NADH regeneration is probably the best approach to achieving this goal.

### 7.4. Photochemical NADH Regeneration

**Figure 12:**
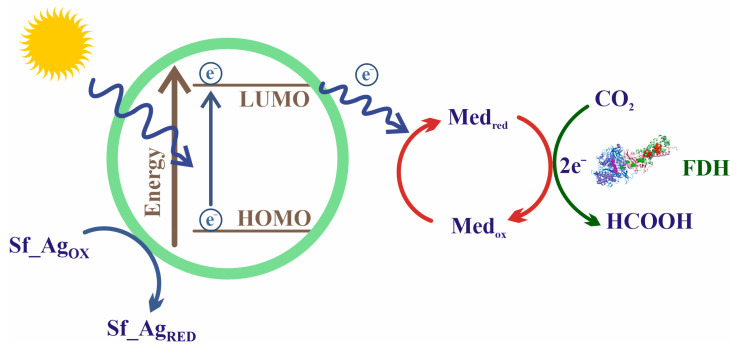
Scheme of the photochemical reduction of CO_2_ using FDH.

Photochemical reactions are another suitable approach to reducing CO_2_ [31,229,230,231,232]. The energy arises from light, which is an inexpensive, renewable, and clean source. This requires a supply of electrons and a photosensitizer mediator scheme (Figure 12). The electron donor, *D*, also called the sacrificial agent, is typically a stable solute present in large concentrations that can be easily oxidized using a photosynthesizer in its excited state [223]. Amines such as triethanolamine (TEOA), triethylamine (TEA), and ethylenediaminetetraacetic acid (EDTA) are the most commonly employed, although many other species, such as 3-(*N*-morpholino) propanesulfonic acid, 4-(2-hydroxyethyl)-1-piperazineethanesulfonic acid, 2-(*N*-morpholino)ethanesulfonic acid mercaptoethanol, phosphite, propanol, and even molecular hydrogen gas have also been used [30]. The redox potentials of these systems must be low enough to be oxidized using the photosynthesizer so that a pool of electrons is always present in the solution. This electron donor supplier must be present at high concentrations, typically not lower than 100 mM, for the system to be effective. A water molecule has also been proposed, imitating natural photosynthesis, as a sacrificial agent; however, the redox potential of H_2_/H_2_O is too low to produce an efficient system.

The key to these reactions is photosensitizers, molecules that are excited by light [31,232]. These species act as electron mediators, capturing electrons from the sacrificial agent and transferring them to the cofactor (Figure 12). Then, the cofactor is regenerated, and CO_2_ can be reduced using FDH. The mediator is a molecule that is stable in its oxidized state and can be photoactivated, passing from the ground to an excited state in which this molecule has a much higher redox potential, being able to oxidize the sacrificial agent and becoming reduced. In this reduced state, the mediator is highly reducing and immediately relinquishes the electrons to the cofactor. This transfer proceeds with a proton cession, such that the cofactor in its oxidation state (NAD^+^ or others) is reduced (NADH or analogous). The photosensitizers must fulfill three requirements [30,264]: First, the band gap between the HOMO and LUMO must be low enough to accept an electron from visible light (see Figure 3). This is normally satisfied in extended π–π-conjugated systems or semiconductors. Second, in the excited state, its redox potential must be sufficiently high to oxidize the sacrificial agent under solution conditions. Finally, the ground state redox potential must be sufficiently low to reduce the cofactor. Other chemical (stability), economical (inexpensive), and environmental (clean) requirements must also be satisfied.

Various photosensitizers have also been used. Rh(III) and Ru(III) bipyridyl complexes are well known for their photosensitivity; consequently, their use has been extended [264,265,266,267,268,269]. Guo et al. employed Cp*Rh(bpy)(H_2_O)]^2+^ (Cp = cyclopentadienyl; bpy = 2,2′-bipyridyl) as a synthesizer in a system where FDH and FalDH were immobilized on polyethylene membranes doped with the widely used semiconductor TiO_2_ [270]. A comparison between the results using water or EDTA as a sacrificial agent and as a function of pH was described, the latter being much more efficient for formaldehyde production. The optimal FDH:FalDH ratio was 1:0.3, reaching up to 6.5% formaldehyde production after 4 h of reaction. Photosynthesizers composed of xanthene dyes have also been used. Kim and col. combined eosin Y with cobaloxime complexes for regenerating NADH using TEOA as the sacrificial electron donor [271]. The system exhibited an acceptable turnover number for formate generation (*ca.* 1.6) and an optimal NADH production (0.038 mM/h). In situ changes in eosin Y infrared and UV-visible spectroscopy properties eosin Y was also used to follow in situ formic acid generation using infrared and UV-visible absorption spectroscopies [272]. Interestingly, EDTA was used not only as an electron sacrificial agent but also as a source of CO_2_, without any additional electron carriers.

Nanomaterials, which act as porphyrin-based photosensitizers, are another set of well-developed approaches for acting in these photochemical reactions. Ji and coworkers designed a biomimetic chlorosome by combining porphyrin, eosin Y, and [Cp*RhCl_2_]_2_ to generate supramolecular assemblies [273]. Using TEOA as the ultimate electron donor agent, an enzyme cascade was coupled, obtaining 38 μM methanol from CO_2_ after 2 h of reaction. MOFs have revolutionized many technochemical applications [157]. The MOFs used for CO_2_ reduction are also basic pivots in this respect. MOFs contain molecules and holes with different chemical and physical features (hydrophilicity, hydrophobicity, acid-base properties, photochemical features, etc.) that make them ideal for catalysis in general, and photobiocatalysis in particular. An excellent recent example is Xing’s work [229], where a porphyrin ligand was covalently bound to a Zr-based MOF and, posteriorly, the complex Cp*Rh(bpydc)Cl_2_ (bpydc = 2,2′-bipyridine-5,5′-dicarboxylic acid) was incorporated into the organic frame, generating a system that can be activated by light owing to the porphyrin, the Rh(III) complex, and original aromatic ligands of the MOF. FDH was then immobilized via electrostatic entrapment. Using TEOA, up to 244 μg/mL of formic acid was formed after 4 h of reaction. A similar approach employing an MOF based on pyrane skeleton linkers, NU-1006, has also been developed [261]. In this case, the Rh(III) complex attached to the MOF was used as an electron mediator, reducing NADH and being reduced by a pyrene photosynthesizer. FDH was also trapped in the MOF and the reaction took place with a yield of formic acid production of 0.144 ± 0.003 mM after 24 h, while the cofactor was regenerated at a rate of 28 mMh^−1^. In another recent study, MOF Mil-125-NH_2_ was functionalized with the Rh complex in such a way that it was covalently fixed to the secondary sphere of the MOF and FDH was subsequently immobilized [274]. The system obtained a formic acid yield of 9.5 mM in 24 h, whereas the NADH regeneration was 64%.

Graphitic carbon nitride (g-C_3_N_4_)-based materials are other fascinating agents with extremely interesting properties, such as photocatalysts [275]. These materials have also been used in biocatalysis and to generate formate from CO_2_. Zeng et al. used g-C_3_N_4_ doped with the metal dichalcogenide WS_2_, which provides g-C_3_N_4_ with specific semiconductor properties that can be used as photosensitizers [276]. Using [Cp*Rh(phen)H_2_O]^2+^ (phen = 1,10-phenanthroline) as a mediator and TEOA as the sacrificial agent, a multicascade system for obtaining methanol from CO_2_ was designed with acceptable results (methanol productivity 372.1 μmol h^−1^ *g*cat^−1^). In a similar approach, Meng and col. designed nanospheres with thiophene incorporated into hollows in a double shell that acted as the photosynthesizer [277]. This nanomaterial was coupled to [Cp*Rh(bpy)H_2_O]^2+^, which in turn was coupled to the cofactor to reduce CO_2_. Optimal NADH yield regeneration was obtained (74%). Silver nanoclusters combined with TiO_2_ and g-C_3_N_4_ have also been employed in efficient devices for formate production [278]. These nanoclusters are good light sensors, making it possible to obtain good yields in CO_2_ uptake with the metal-dependent FDH from *Clostridium ljungdahliia*. Carbon nitride has also been used in microbial electrosynthesis. Here, the photoanode chamber was formed using an activated carbon fiber (ACF) supported by NiCoWO_4_ in g-C_3_N_4_ [279]. The oxidation of water by light in this chamber provides the electrons for reducing CO_2_ in the cathode chamber, formed by a g-C_3_N_4_/ACF (without NiCoWO_4_), in which a culture of *E. coli* was adhered as a biofilm. FDH from *E. coli* produced the reduction of CO_2_ due to the electrons arriving from the biocathode and the protons, also produced in the cathode by the photochemical decomposition of water, which crossed a cation exchange membrane. The witty system, first applied in CO_2_ reduction, provided highly efficient formate synthesis (12.8 mM per day).

When dealing with photochemical reactions, a critical point is the reactive oxygen species (ROS) that can be generated by photoexcitation processes. To avoid this, systems mimicking chloroplasts have been developed, creating divided compartments where reactions occur separately. The photoactivation process was achieved by combining a Rh(III) complex conjugated onto g-C_3_N_4_ previously modified using thiophene, with triethanolamine used as the electron sacrificial agent [280]. The key to this approach arises from the encapsulation of FDH into a MOF (MAF-7), which protects the enzyme from photoactivation reactions. NADH shuttled electrons from the reduction compartment towards FDH, obtaining 16.75 mM of formic acid after 9 h of illumination. Immobilization of FDH together with CA in the TOF ZIF-8 was also carried out by Yu et al. in another system imitating photosynthesis and with g-C_3_N_4_ as the photosynthesizer [281]. CA accelerated the interconversion of CO_2_/HCO_3_^−^, allowing for an effective mass transfer rate between the gas and liquid phases. The authors reported production of 243 μM formic acid with excellent system stability since the yield production was above 80% after 10 batches.

Graphene has also been employed as a photocatalyst in systems that imitate photosynthesis and produce formic acid from CO_2_ [210]. Ji’s group conceived and developed a nanofiber polyurethane system as a support where the cationic electrolyte, a polyalanine, was deposited onto graphene oxide, while the cascade of proteins FDH, FalDH, and ADH was entrapped in the hollows of the nanofiber to generate methanol [246,282,283]. Finally, polymers that mimic photosystems in CO_2_ reduction have also been candidates for biotechnological applications. Kim et al. reported a photosystem based on polydiacetylenes and covalently attached (phen)Ru(bpy)_2_ as the mediator [284]. The system exhibited acceptable regeneration values for NADH.

It should also be noted that semiartificial photocatalysis centers have been also designed to reduce CO_2_. Sokol et al. generated a sophisticated electrocell in which the photoanode was the photosystem II from *Thermosynechococcus elongatus* embedded in a redox polymer composed of the complex [Os(bipy)_2_Cl]Cl bound to a polymer of a derivative of allylamine was, in turn, deposited on TiO_2_ [285]. Here, light (680 nm) oxidized water to O_2_, generating electrons that were shuttled towards the cathode, where FDH from *Desulfovibrio vulgaris* had adhered to TiO_2_ but, in this case, coated with a fluorine tin oxide. Excellent Faraday efficiencies (>70%) and acceptable yields (0.185 μmol/cm^2^) were obtained, although the system exhibited the progressive photodegradation of PSII.

Due to its high substrate and product selectivity as a biocatalyst, FDH has been used in recent years to obtain chemical products from reduced forms of CO_2_, such as formic acid. In addition to its own applicability, this acid is used as a springboard product to obtain a wide variety of derivatives. For example, CO_2_ can be converted to oxalic acid through the coupling of two formate molecules [286]. This can occur via a metal–formate intermediate (typically sodium or potassium alkaline metals), obtained from the electro- or photocatalytic reduction of CO_2_ coupled to FDH. After obtaining this metal–formate, two molecules are coupled to give oxalate, which, after acidification, gives rise to oxalic acid. Oxalic acid produced through this coupling route can be used in various industries, including pharmaceuticals, as a component of some antibiotics, or textiles and in other industries such as food (beer or wine production) or chemicals [286]. In addition, from the reduction of this oxalic acid, a wide variety of derivatives are obtained. The first oxalic acid reduction product is glyoxylic acid, which, after reduction, produces glycolic acid and also ethylene glycol. These chemicals are the starting points in the production of agrochemicals, flavors, cosmetics, and polymers [287].

### 7.5. CO_2_ Reduction by Whole-Cell Bacteria

Finally, it should be pointed out that recent studies focused on CO_2_ reduction encompass an even more open overview of what nature provides us in this area. Indeed, whole cells can also be used for fixing CO_2_, using H_2_ as the reductant agent [288,289,290], electrochemical reduction [291], or even a combination of H_2_ and photocatalysis [290]. Whole-cell biocatalysis is a promising method developed in the last few years that shows high efficacy and selectivity and can be used in soft conditions. Moreover, since there is no requirement for purifying enzymes, the most expensive step in biocatalytic processes, the whole-cell approach is a hopeful methodology that will probably have the highest efficiency/cost ratio. It is also relatively easy to adjust the experimental conditions to be developed if the suitable organism or correct molecular biology tools in them are adequately employed. Table 5 reports the most relevant results obtained in the CO_2_ reduction to formate using the whole-cell approach. The culture of these microorganisms supplied by H_2_ gas reduces CO_2_ obtaining extraordinarily high concentrations of formate. Methylobacteria species are satisfactory in this aspect [290].

Leo et al. genetically modified *E. coli* cells to express the hydrogen-dependent CO_2_ reductase from *Acetobacterium woodie* [294]. In this bacterial culture, increasing the cell density up to 30 mg/mL resulted in a formate production yield of 6 mM/h, whereas the addition of 2.5 mM HCO_3_^−^ increased the formate generation rate fourfold. In this way, the authors confirmed that *E. coli* CA also played an important role in providing the correct substrate to FDH. Müller’s laboratory took advantage of the metabolic machinery of the acetogenic bacteria *Acetobacterium woodii* and *Thermoanaerobacter kivui* to convert H_2_ and CO_2_ from syngas into formic acid, obtaining production rates of 234 mmol g^−1^_protein_ h^−1^ [288]. *E. coli* has been also used for formate generation with and without overexpression of exogenous FDH genes. Indeed, as commented previously (see Section 3.2), *E. coli* has two metal-dependent FDHs capable of reducing CO_2_ to formate. This bacterium has been used in the whole-cell strategy with extraordinarily high formate generation. Indeed, more than 0.5 molL^−1^ of formate concentration was achieved when *E. coli* cells were grown under CO_2_ and H_2_ gas at 10 bar pressure for 23 h [293]. *E. coli* JM109(DE3) strain has also been used as a host bacterium where to overexpress FDHs from *Clostridium carboxidivorans, Pyrococcus furiosus (Pf)* and *Methanobacterium thermosformicicum,* also obtaining excellent yields [292]. Indeed, the highest formate generation yield of this study, obtained with *Pf*FDH, was more than 1 gL^−1^h^−1^.

Electrochemical reduction is another approach for obtaining high yields of formate within the whole-cell frame. An excellent and very recently released review describes in detail this methodology [291]. Table 5 highlights the remarkable features of the—up to now—few articles published in this area concerning formic acid generation. In these studies, yields are as good as for those using the H_2_ reduction strategy. For instance, Le et al. achieved to obtain 3.8 mM h^−1^ g^−1^_wet-cell_ of formic acid in the cathode electrode at 72 h when *Shewanella oneidensis* MR-1 was grown in LB media supplemented with nitrate 1 mM and DL-lactate 20 mM [296].

It is also remarkable that two other different approaches to applying whole-cell have been developed. Guntermann and col. devised a two-phase system where *Saccharomyces cerevisiae* D-glucose fermentation produces ethanol and CO_2_ in the water phase. This was coupled to a hydrogen gas source that, in the presence of a Ru catalyst solved in the tetradecane phase, generated formic acid at concentrations of 128 mM after 48 h of reaction [300]. On the other hand, light-driven photocatalytic hydrogenation has been applied to a culture of *Shewanella oneidensis* MR-1 in anaerobic conditions [301]. Using methyl viologen as a photoactivated molecule and triethanolamine (TEOA) as the sacrificial agent, Rowe et al. obtained formic acid at concentrations higher than 1.5 mmol for 48 h of reaction.

Finally, it is worth remarking that the use of microorganisms that incorporate formic acid into their metabolic routes has also been another object of research in the last years. Formatotrophic organisms (capable of assimilating formate for use as a carbon source) offer a new approach to formate utilization, although they are difficult to cultivate, which partially restricts their applicability [302,303]. One strategy is to biotechnologically adapt microorganisms to assimilate formate by adapting their metabolism to the formatotrophic growth model through metabolic engineering tools. Both methanol and formate can be assimilated in the central metabolism through various metabolic pathways, and the bio-production of other compounds such as ethanol, acetone, isopropanol, or short- and medium-chain fatty acids and alcohols from these compounds is very promising [304,305]. To this end, the introduction of pathways, such as the Calvin cycle, the serine cycle, the acetyl-CoA reductive pathway or the glycine pathway in hosts, or the design of new pathways, is proposed [304,305]. The most commonly used hosts are *E. coli*, *S. cerevisiae* and *Cupriavidus necator*, as they are easy to modify with genetic engineering tools and are industrially applicable [306].

Synthetic pathways, such as the reductive glycine pathway (rGly, pathway for formate assimilation), can support higher biological yields than natural pathways and could therefore be implemented in a variety of microorganisms. The rGly pathway has been introduced into the *E. coli* host by redesigning its central metabolism to be able to assimilate formate. In terms of strategy, the pathway to be integrated is divided into modules and introduced together into the host bacteria to express the enzymes necessary to form the complete metabolic pathway [304,305]. With this methodology, a strain capable of growing from formate with a doubling time of ~70 h and a growth yield of ~1.5 g cell dry weight (gCDW) per mole of formate was achieved [303]. In this way, products such as lactate or isobutanol, both pyruvate derivatives, were obtained. Cotton et al. used *C. necator* to modify it with rGly, so that it was able to grow under formate-rich conditions. In this respect, the production of methylketones, isoprenoids and terpenes, isobutanol, alkanes, and alkenes from CO_2_ using *C. necator* seems particularly promising. With the same microorganism, the Calvin cycle was also used for the assimilation of formate or methanol, although with low energy efficiencies (20–35%) [307]. On the other hand, Collas et al. very recently devised an intelligent approach in which they engineered a crotonate biosynthetic pathway in *C. necator* [306]. This mechanism permits the conversion of formic acid in crotonate with extraordinary yields in a continuous process. Indeed, using this approach, they obtained 148 mg/L of product. This is a new starting point for the generation of new value-added chemicals.

Although whole-cell technology is still incipient, new opportunities that open a window to capture CO_2_ under standard conditions, where nature develops, are enormous.

## 8. Conclusions and Perspectives

A panoramic overview of the state-of-the-art on carbon capture and its reduction in C_1_ forms by employing the biocatalysts CA and FDH was presented. CO_2_ uptake is one of the main challenges that science, in general, and chemistry, in particular, face today. Nature offers appropriate tools and clues for finding approaches to solve this huge problem posed to mankind. CCSU techniques are well developed in the laboratory and, to a lesser extent, at a large scale, although their application is still far from being appropriately adapted for obtaining high yields without great energetic costs. While CA immobilization is currently a reality and it stabilizes the enzyme allowing its reuse, the employment of recombinant CAs with higher resistance to temperature and pressure is a field that still remains to be fully developed. These molecular biology tools will help speed up CO_2_/HCO_3_^−^ conversion at an industrial scale in the next few years.

Carbon dioxide reduction is also a reality at the laboratory scale, and research lines are well established, although they are still far from showing high yields. The formic acid molecule is a rich form for transporting hydrogen in an efficient way and is the primary step for producing many more reduced molecules in such a way that carbon is recycled and reused as an energy H_2_ vector. Mass transfer from the gas to the solution is a limiting step in reaching the substrate to the biocatalyst. On the other hand, improvements in the stability and high performance of FDHs, as well as in their stability and reuse via immobilization, have been developed, with a significant explosion of this research in the last decade, despite still being a relatively virgin area. However, its application to larger scales has not been established.

Measures to mitigate climate change are urgently needed. CAs and FDHs are excellent devices for capturing CO_2_ and transforming it into fuel, which is an interesting way to reduce two problems into one. According to the exponential progress existing in the research in this area nowadays, surprising approaches and solutions to these problems will probably be found not in the next decades but in years.

## Figures and Tables

**Scheme 1:** Acid–base equilibria for CO_2_ water-soluble species as a function of the pH (Equations (1)–(4)) and reduction CO_2_ equation to generate formic acid using the cofactor NADH (Equation (5)).

**Scheme 2:** Reactions of CO_2_ with primary, secondary (Equation (6)), and tertiary (Equation (7)) amines in water solutions.

**Table 1:** Features of representative carbonic anhydrases.

Type	Organism	MW (kDa)	Activity (WAU/mg)	Activity (*k*^−1^)	Metal Coordination	Ref.
α	Bovine	29.8	2540		Zn(II), 3 His, H_2_O	[53]
	*Homo sapiens* (HCAI)	28.7	920	2.0 × 10^5^		[54]
	*Homo sapiens* (HCAII)	29.1	8000	1.4 × 10^6^		[54]
	*Persephonella marina*	26.9	1748			[55]
	*Thermosulfurimonas dismutans*	27.9	2032			[53]
	*Thermovibrio ammonificans*	25.3	1016			[53]
	*Bacillus halodurans*	37.0	3425			[56]
	*Sulphurihydrogenibium yellowstonense*	26.0	7254			[57]
β	*Bacillus subtilis*	37.0	714		Zn(II), His, 2 Cys	[58]
	*Acetobacterium woodii*	22.0	1814			[55]
	*Methanobacterium thermoautotrophicum*	19.9	580			[55]
	*Aspergillus fumigatus*	23.0	20			[59]
γ	*Geobacillus kaustophilus*	22.0	179		Zn(II) or Fe(II), 3 His, H_2_O	[60]
	*Thermus thermophilus HB8*	24.3 ^a^	0.9			[61]
	*Methanosarcina thermophila*	40.0	4872			[62]
	*Burkholderia pseudomallei*	28.2		5.3 × 10^5^		[63]
	*Vibrio cholerae*	26.3		7.39 × 10^5^		[63]
	*Porphyromonas* *gingivalis*	26.2		4.1 × 10^5^		[63]
δ	*Thalassiosira weissflogii,* TWCA1	27.0		1.3 × 10^5^	Zn(II), 3 His, H_2_O	[64]
δ	*Emiliania huxleyi*	18.3		1.3 × 10^6 (b)^	Zn(II), 3 His, H_2_O	[65]
ζ	*Thalassiosira weissflogii,* CDCA1	69 ^a^		1.5 × 10^6^	Cd(II) or Zn(II), His, 2 Cys	[42,66]
η	*Plasmodium falciparum*	26.2		1.4 × 10^5^	Zn(II), 2 His, Gln, H_2_O	[43,67]
θ	*Thalassiosira* *pseudonana*	26.0	122		Cd(II) or Zn(II), His, 2 Cys	[38]
	*Phaeodactylum tricornutum*	31.1	30.9			[47]
ι	*Burkholderia territorii*	28.2		3.0 × 10^5^	Cd(II) or Zn(II), His, 2 Cys	[46]
	*Anabaena* sp. PCC 7120 ^c^	19.3		16.7		[52]
	*Bigelowiella natans* ^d^	55.3		85.8		[52]

^a^ A trimer formed by three chains, R1, R2, and R3. ^b^ CA activity in the cytoplasm. ^c^ Activity of the GOG4337 recombinant protein all2909 (ref. [52]). ^d^ Activity of the GOG4337 recombinant protein Bn86287 (ref. [52]).

**Table 2:** Kinetic and thermodynamic parameters of most relevant FDHs, classified into their two groups. Molecular weights and pH conditions in which these conditions were determined are also indicated. For metal-dependent FDHs, the types and number of their subunits as well as the cofactors of their active centers are also indicated.

**A. NAD-Dependent FDHs.**												
			**Formate Oxidation**				**CO_2_ reduction**					
**Organism**		**MW (kDa) ^a^**	**pH**	***k*_cat_ (s^−1^)** **(U mg^−1^) ^b^**		***K*_M_ (mM)**	***k*_cat_/*K*_M_** **(mM^−1^ s^−1^)**	**pH**	***k*_cat_ (s^−1^)** **(U mg^−1^)**	***K*_M_ (mM)**	***k*_cat_/*K*_M_** **(mM^−1^ s^−1^)**	**ref**
*Myceliophthora thermophila*		42	10.5	0.32		7.2	0.04	7.0	0.10	0.43	0.23	[97]
*Ancylobacter aquaticus*		45	6.0	(21.6)				6.0	(23)		4.5	[98]
*Candida boidinii*		41	7.0	1.081 (6.1)		8.55	0.13	5.5	0.015	2.6	0.006	[98]
*Thiobacillus sp.* KNK65MA		45	6.5	1.769 (10.9)		16.24	0.11	5.5	0.32	0.95	0.34	[98]
*Candida methylica*		42	8.0	1.31 (13.2)		7.01	0.19	8.0	0.008	0.078	0.01	[97]
*Chaetomium thermophilum*		45	5.0	2.04(3.1)		3.30	0.62	5.0	0.023	3.29	0.069	[93,99]
*Ceriporiopsis subvermispora*		40	6.5	(1.3)				6.0	(0.8)			[98]
*Moraxella sp.* C-1		45	5.5	(14.3)				5.5	(2.8)			[98]
*Paracoccus sp.* 12-A		45	5.5	(12.2)				5.5	(6.5)			[98]
**B. Metal-Dependent FDHs**												
				**Formate Oxidation**				**CO_2_ reduction**				
**Organism**	**MW ^a^ (kDa)**	**Subunits ^b^**	**Cofactors**	**pH**	***k*** **_cat_** **(s^−1^)** **(U mg^−1^)^a^**	***K*** **_M_** **(mM)**	***k*** **_cat_** **/*K*_M_** **(mM^−1^ s^−1^)**	**pH**	***k*** **_cat_** **(s^−1^)** **(U mg^−1^) ^a^**	***K*** **_M_** **(mM)**	***k*** **_cat_** **/*K*_M_** **(mM^−1^ s^−1^)**	**ref**
*Syntrophobacter fumaroxidans* FDH-1	175	(αβγ)2	W, SeCys 4 [Fe2S2] [Fe4S4]	7.0	(700)	0.04	-		282 (900)	-	-	[100]
*Syntrophobacter fumaroxidans* FDH-2	125	(αβ)2	W, SeCys 2 [Fe2S2]	7.0	(2700)	0.01	-		282 (89)	-	-	[100]
*Desulfovibrio desulfuricans*	135	(αβγ)3	Mo, SeCys 2 MGDs 4 *c*-heme 2 [Fe4S4]	8.0	543	0.0571	9526	7.0	46.6	0.0157	2968	[101]
*Escherichia coli* FDH-H	79	αβ	Mo, SeCys 2 MGDs 1 [Fe4S4]	7.5	2800	26	107.7	7.5	1.0	8.3	0.12	[102,103]
*Desulfovibrio vulgaris* Hildenborough	97.4	(αβχ)3	Mo, SeCys 4 [Fe4S4] 4 *c*-heme	7.6	1310 (77)	0.017	77.06		315(1.0)	0.42	750	[104,105]
*Acetobacterium woodii*	169	(αβ)3	Mo SeCys [4Fe-4S])	7.0	(600)	1.0	-	7.0	372 (132)	3.8	97.9	[106]
*Cupriavidus necator*	178	(αβγ)3	Mo, 4 [Fe4S4] FMN 3 [Fe2S2]	7.0	140	0.082	1707	7.0	11	2.7	4.07	[107]
*Rhodobacter capsulatus*	180	(αβγ)2	Mo, 4 Fe4S4, 1Fe2S2, 2 MGD, FMN	5.0	36.5	281	0.13	7.7	1.48	-	-	[108,109]
*Pseudomonas oxalaticus*	315	-	Mo, 2 FMN		-	0.135	-	6.2	3.0	40	0.075	[110,111]
*Clostridium Ijungdahlii*	80	-	W Cys 2 MGD [Fe4S4]	9.0	14.77	1.40	10.55	7.0	0.73	7.27	0.17	[112,113]
*Clostridium autoethanogenum*	74		W 2 MGD [Fe4S4]	9.0	1.04	4.51	0.231	7.0	4.00	23.15	0.17	[113]
*Clostridium coskatii*	62		W 2 MGD [Fe4S4]	9.0	0.62	5.57	0.111	7.0	5.62	59.65	0.094	[113]
*Clostridium ragsdalei*	74		W	9.0	11.88	44.83	0.265	7.0	3.28	31.20	0.11	[113]
*Desulfovibrio gigas*	121	(αβ)2	W, SCys 4 [Fe4S4]	8.0	(34.1)	-	-	-	-	-	-	[114]
*Moorella thermoacetica*		(αβ)2	W, SeCys 4 [Fe4S4]	7.5	(1100)	-	-	-	-	-	-	[115]

Sub-Table (A): ^a^ Molecular weight of the monomer (most FDHs are associated with dimers). ^b^ Data between parenthesis refer to the activity in units (μmol min^−1^) of formate/CO_2_ oxidized/reduced per mg of FDH; Sub-Table (B): ^a^ Molecular weight of the monomer. ^b^ The number refers to the number of subunits, that is, the composition of dimers or trimers of each FDH. MGD: molybdopterin guanidine dinucleotide. FMN: flavin mononucleotide.

**Table 3:** Immobilization of Carbonic Anhydrase using different methods.

Method	Support	Immobilization Conditions	Main Results	Ref
Physical adsorption	Mesoporous aluminosilicate	Phosphate buffer 100 mM, pH 7, 0.2 mL of enzyme (1 mg/mL) in 10 mg of support for 6 hr at 120 rpm.	CA loading up to 3 mg/mL optimal for CA activity. Carbonation activity was 55% (8 cycles of reuse	[140]
	Carboxylic acid group-functionalized mesoporous silica (FMS)	0.6–2.0 mg FMS with functional groups incubated with 70–1000 IL of 2.0 mg/mL BCA II in water (pH 6.5) for 2 h at 21 °C under 1200 rpm shaking.	High protein loading density 0.5 mg of protein/mg. High stability and activity (95.6%) of the immobilized enzyme	[141]
	Silver nanoparticles amine-functionalized mesoporous SBA-15	10 mg dispersed separately in 2 mL free HCA in buffer (3 mg/mL HCA in 100 mM sodium phosphate, pH 6.4) followed by incubation at 25 °C with shaking for 4 h.	The activity of the materials was ∼25-fold higher than the activity of the free HCA for converting CO_2_ to CaCO_3_ after 30 cycles.	[142]
	Mesoporous silica nanoparticles with polydopamine (PDA) and polyethyleneimine (PEI)	5 mg FDH/1.6 mg CA added to 2 mL of phosphate buffer (0.05 M, pH 6). PDA/PEI-mSiO_2_ (0.05 g) and 5 μL GA aqueous solution (20 wt%) were added (30 °C for 24 h).	Optimal FDH and CA concentration was 2.5 and 0.8 mg/mL respectively, (specific activity of 0.045 mM/h/mg). 10 reuse cycles with 86.7% of activity.	[143]
	Palmityl-substituted sepharose 4B	Denatured Bovine CA solution by incubation for 15–120 min (58–65 °C), Tris-sulfate buffer, pH 7.5 (0.0125–0.5 mg/mL).	Denaturation and renaturation processes of the enzyme in the matrix. After renaturation at 60 °C, 85% of immobilization was achieved (70% of the original activity after 1 h).	[144]
	Silica or titania particles	The clarified lysates expressed BCA-peptide (0.088 mg protein) were incubated with 30 mg silica or 20 mg titania for 10 min with shaking at room temperature in 25 mM sodium phosphate buffer (pH 6.5).	After 10 days, 90 ± 4% and 95 ± 3% of silica and titania particles’ original activity remained. Immobilized BCA-peptide fusion protein shows ∼95% of its residual activity with up to 5 cycles of reuse.	[145]
	Porous polypropylene and a non-porous polydimethoysilane hollow fiber membrane	Application of CA in situ to the shell side surface of each fiber.	CO_2_ absorption into K_2_CO_3_ increased approximately three-fold when CA is adsorbed onto the Porous polypropylene or non-porous polydimethoxysilane PDMS membrane surface	[146]
Entrapment or encapsulation	Magnetic nanoparticles (sol-gel ferria hydrosol)	200 mL of ferria hydrosol mixed with CAB solution (10 g/L, 0.05 M Tris, pH 7.4) and dried at 20 °C.	Large thermal stability of CA immobilized, active up to 95 °C	[147]
	Polyurethane foam (PUF)	1 mL of CA (1 g/L) or whole-cell solution in 20 mM Tris-sulfate buffer (pH 8.3) was added to a 50 mL containing 1 g of prepolymer. The swelling of PUF continued for 30 min, and then an additional 10 min was allowed for curing.	An immobilization efficiency of 3.4%, 16-fold higher than that for free enzymes. The reusability of the immobilized whole-cell catalyst shows no apparent decrease in activity after 9 reuses. The rate of CO_2_ capture was accelerated by 80%.	[148]
	Ni-based MOFs (Ni-BTC) nanorods,	100 mL cell lysate of His-HCA II (0.4 mg protein) and 900 mL Milli-Q water were incubated with Ni-based MOFs.	His-HCA II from cell lysate obtained an activity recovery of 99%. After storing for 10 days, the immobilized His-HCA II maintained 40% activity (free enzyme lost 91% activity). Immobilized His-HCA II retained 65% (8 cycles).	[149]
	Supported ionic-liquid membranes (SILMs)	0.25 mg CA/g IL.	SILMs resent permeability (PCO_2_ = 733.73 barrier) at high temperatures (up to 373 K) and a good transport selectivity towards CO_2_ against N_2_.	[150]
	Cholinium-based ionic liquids	0.1 mg CA/g IL	CA samples promote an enhancement of 63% in the carbon dioxide transport rate.	[151]
Covalent binding and cross-linking	Mesoporous SBA-15 surfaces covalently functionalized with amines	HCA immobilization was achieved by mixing 10 mg of amine-functionalized SBA-15 with a 0.1% glutaraldehyde (GA) solution (50 mM sodium phosphate, pH 8.0, 1 h). The product was treated with free HCA (3 mg/mL HCA, pH 7.0), incubated and shaking (1 h, 25 °C).	Immobilized HCA retained activities after long-term storage, exposure to high temperatures, and reuse (40 cycles). CO_2_ capture efficiency of immobilized HCA was 36 times higher than that of free HCA, 75% of the enzymatic activity was retained after 40 cycles.	[152]
	Chitosan	CA liquid was slowly added into the pH 5 chitosan solution with continuous stirring. Ratios of 1:0.05–1:2 chitosan:CA (g:mL)	Textile packing with covalently attached enzyme aggregates retained 100% of the initial 66.7% CO_2_ capture efficiency over 71 days and retained 85% of the initial capture efficiency after 1-year of ambient dry storage.	[153]
	Polyethyleneimine and polydopamine in MOF 808	PBS buffer (pH 8, 10 mM), PEI/PDA-MOF-808 or PDA-MOF-808 was disseminated. 200–400 μL of CA solution (1 mg/mL, Milli-Q) and 10 μL of aqueous solution of GA (0–25 wt%) were added and shaken for a while at 28 C.	CaCO_3_ produced by CA@PEI/PDA-MOF-808 was 11.0-fold and 2.5-fold higher than free CA and PEI/PDA-MOF-808, respectively. After 8 consecutive rounds, the total production of CaCO_3_ by CA@PEI/PDA-MOF-808 was 92-fold higher than free CA.	[154]
	Alumino-siloxane hybrid aerogel beads	100 mg of Al/Si-NH_2_ beads were treated with 0.5% GA for 1 h. 4 mL of free BCA in the buffer (1 mg/mL 100 mM phosphate buffer) was added to GA-treated Al/Si-NH2 beads and stirred (3 h, 25 °C) for BCA immobilization.	Free BCA retained 70% of its maximum activity (immobilized BCA, 88%). Free BCA and BCA-Al/Si-NH2 remained 80% of the activities, after ten days. BCA-Al/Si-NH_2_ retained 89% of their enzyme activity up to 10 cycles	[155]
	Amine-functionalized by co-deposition of polydopamine (PDA) and polyethyleneimine (PEI)	Co-deposition of PDA and PEI and CA covalently anchored on the surface via GA. Surface was treated with a mixture of PDA (2 mg/mL) and PEI, pH 8,5. Amine-functionalized micro-reactor surface was contacted with a mixture of CA and GA (concentration 2.0% (*v/v*)).	A steady CO_2_ absorption rate for several hours and good reusability of the immobilized enzyme which maintains its original absorption performance after 10 cycles of operation.	[156]
	Polypropylene hollow fiber membranes using GA-activated chitosan	Aminated knitted hollow fiber membranes mats (204 cm^2^ fiber surface area) were incubated in 5% GA in 100 mM phosphate buffer, pH 8.5 for 1 h under constant rocking at room temperature.	Chitosan/CA coated fibers exhibited accelerated CO_2_ removal in scaled-down gas exchange devices in buffer and blood (115% enhancement vs. control, 37% enhancement vs. control, respectively).	[157]
	Magnetic Cross-Linked Enzyme Aggregates (CLEAs) to bovine carbonic anhydrase (BCA) and magnetic nanoparticle	- Adsorption: the NPs suspension (5 and 2 mg solids) added to 1 mL of 10 mM PBS (pH 7.4), 10 g/L BCA. - Precipitation: NP-enzyme suspension added dropwise to 9 mL containing the precipitating agent (pH 7.4), 1 h or 0.5 h under mixing. - Cross-linking: 25% vol GA added. The system kept under mixing for 3, 16 and 22 h. - CLEAs separated by MF.	BCA-CLEAs can increase the CO_2_ absorption rate concerning the one observed in the same reactor filled with only alkaline solvent. Biocatalyst reusability analysis showed that BCA-CLEA retained 95% of its initial activity after five CO_2_ absorption tests at 1000 mg BCA CLEA/L and as many liquid-solid separation steps by membrane filtration.	[158]
	Geopolymer micro-spheres (GMS) and covalent attachment by GA	GMS were introduced into a Tris-HCl buffer solution (0.05 M, pH 8.0) with CAs solution (1 mg/mL) in a 50 mL centrifuge tube. After shaking and reacting, the GA was added for the cross-linking between the GMS and CAs.	*K*_cat_/*K*_m_ values were 61.50 and 12.36 M^−1^s^−1^ for the immobilized and free CAs, respectively. At 60 °C, free CAs were inactivated (immobilized CAs kept 34.8% activity). Immobilized CAs maintain 68.73% of activity (8 cycles).	[159]
	Microbial transgluta-minase (MTG) acts as a “cross-linking medium”	Iso-peptide bond between glutamine and the primary amine group of a lysine in artificial peptide tags. Equimolar amounts F-CA and M-FDH were added, and a metal constant temperature oscillator was used for the cross-linking reaction (reaction time 12 h, 25 ℃, 400 rpm. The amount of MTG used was 1 U/mL.	The remaining CA activity was more than 93%, and the remaining FDH activity was more than 84%. The efficiency of the cross-linked enzyme is increased by 5.8 times compared with free enzymes. FDH thermal stability at different temperatures is improved the optimal found CA/FDH ratio was 1:2.	[160]

**Table 4:** Relevant studies on CO_2_ uptake processes performed using CA, and their main features.

Process	Idea of the Study	CO_2_ Uptake Conditions	Relevant Conclusions	CA Activities	Ref.
Chemical absorption	To examine the stability and activity of the enzyme under different pH values and amine solvents. Compare its performance to other common solvents.	CA tested for pH [7,8,9,10,11]. Temperature stability for up to 100 h of incubation. CA stability tested for 7 capture solvents (1 M or 3 M, 150 days, 40 °C).	CA stable at 60 °C, pH range [7,8,9,10,11]: residual activity at pH 5 or 12, ranging from 12 to 91%. The enzyme enhances reaction rates. NaCl, K_2_CO_3_, AMP, and MDEA show additive effects.	After 100 h (25 °C), CA activity was kept higher than 75% for 1M NaCl, AMP and MDEA and 125% in 1M K_2_CO_3_	[191]
	To develop the CA-MOFs composite, with superior catalytic performance and high stability to promote CO_2_ absorption into a tertiary amine solution.	0.05 g L^−1^ and 40 °C, P_CO2_ 15 kPa.	CA loading in ZIF-L-1 increased with the added enzyme amount. CA/ZIF-L-1 composite has higher catalytic activity and stability than the free CA. The immobilization of CA on ZIF-L-1 improves the CA conformational stability	CO_2_ absorption rate of CA/ZIF-L-1 in 1 M MEA and MDEA at 40 °C: 3.0 × 10^6^ kmol s^−1^m^−2^. CA loading reached up to 87 mg g^−1^. The highest immobilized CA activity was 1.5 times that of the free CA	[192]
	Pilot-scale experiments with CA-enhanced MDEA for CC, bench-marking its mass transfer performance against the industrial standard 30 wt% MEA.	Experiments were done using 30 wt% MEA and MDEA solvents varying CA concentrations (0, 0.85, and 3.5 g/L) at different column L/G ratios.	Enzyme-enhanced MDEA solutions exhibit 80% of the mass transfer performance at 30 wt% MEA and have the potential to reduce the size and cost of absorbents in carbon capture.	CO_2_ capture efficiency higher than 90% for high L/G ratios. The mass transfer increased 20-fold for 25 and 50 wt% MDEA solutions by adding 0.2 g/L CA.	[193]
	Use of columns with membranes contractors with polyionic liquids (PIL), amines and CA for CO_2_ uptake.	PIL blend (F9:1(M10)) with immobilized CA in 30 wt% MDEA at low feed gas (15% CO_2_ in N_2_.) pressure (1.3 bar)	Addition of the enzyme to the MDEA solution with PILs significantly improves CO_2_ uptake rate and reduced the equilibration time.	CA addition to MDEA solution improved the CO_2_ uptake rate by 1.7 times. CA and membrane improved the uptake rate by twice.	[194]
Carbonation	To present the effects of adding small quantities of immobilized CA on the absorption of CO_2_ into potassium carbonate.	CO_2_ absorption (partial pressure 90 kPa) in a 30% K_2_CO_3_ solution. A wet-walled column (40–80 °C) with 38 g/L CA.	CA addition improves the CO_2_ absorption in K_2_CO_3_ solvents. The rate of CA-catalyzed CO_2_ hydration increased with the CA concentration	Increasing CA concentration from 0.4 to 1.8 μM increases the absorption rate by 34% (40 °C)	[195]
	To explore the potential of using enzymes to catalyze the conversion of CO_2_ into bicarbonate and on the carbonation rate of brucite, Mg(OH)_2_.	Gas flow (CO_2_/N_2_ 10%/90%) at 10 psig. Experimental durations were 11, 7, and 3 days (low, medium, and high flow experiments).	CA accelerates the carbonation of brucite. Higher CO_2_ gas flow rates results in faster carbonation. Mineralogical compositions depend on the CO_2_ flow rate.	The carbonation rate of brucite using BCA was accelerated by up to 240% compared to controls.	[196]
	To propose a novel method based on microbially induced calcium precipitation to improve the cementitious properties of steel slag.	*Bacillus mucilaginosus* placed in a sealed container, the air pressure was reduced to −0.05 MPa. CO_2_ (99.99%) pumped to maintain the gas pressure (0.25 MPa, 30 °C, 32 h).	Ca-silicate promotes the growth of bacteria, enhances the production of polymers and improves bacterial adhesion. Ca-silicate-containing medium (CSCM) can be used as a microbial carrier.	Maximum bacterial growth rate in CSCM was 1.5 times higher than that in the control medium (CM). Bacterial activity in CSCM was 40% of that in CM (30 h). At a dosage of bacterial powders of 1 wt.%, carbonation reached the maximum.	[197]
	To develop and characterize a highly efficient and stable biocatalyst for CO_2_ sequestration using silica nanocomposite with auto-encapsulated CA. (CA)-based biocatalyst encapsulated in a biosilica with a peptide R5.	CaCO_3_ precipitation was carried out at 30 °C and monitored turbidimetrically at 600 nm. The final pH of the buffer was approximately 9.3	The encapsulated CA was not leached from the silica matrix. Encapsulation in silica effectively improved the thermostability and activity of the enzyme.	Encapsulation efficiency greater than 95%. Residual activity of the self-encapsulated CA was more than 50% (30 min, 80 °C). Activity of ngCA-R5@silica decreased only after 2.5 days at 60 °C. Encapsulated CA: 98% and 80% of its initial activity (1 day and 5 days of incubation, 50 °C). CaCO_3_ precipitation is reduced by 5.5-fold when ngCA-R5@silica (30 μg/mL) is present compared to the uncatalyzed reaction.	[198]
Mineralizaton	Mineralization experiments were performed using *Curvibacter lanceolatus* strain HJ-1, including its secreted extracellular polymeric substances (EPS) and CA (CA).	Three types of mineralization experiments: with CA (duration 96 h), with EPS (96 h), and with bacteria (50 days).	Strain HJ-1, EPS, and CA promote carbonate precipitation. HJ-1 and EPS1 experiments contained calcite and aragonite. CA formed calcite only. HJ-1 and EPS are favorable for aragonite precipitation.	The mass of precipitate (inorganic plus organic substances) increases until ca. 55 mg. The maximum degree of calcification was approximately 6.6%. In the control groups without CA no precipitate was formed. In the absence of HCO_3_^−^, the optimized calcification rate followed the order: HJ-1 (49.5%) *>* CA(6.6%) *>* EPS2(4.1%).	[199]
	Production of CA from the bacterium *Aeribacillus pallidus* TSHB1, a thermostable and alkaline-stable bacterium, highly effective in the formation of CaCO_3_ from aqueous CO_2_.	Tris buffer (15 mL, pH 7.4) containing 0.9 g CaCl_2_-2H_2_O, CA 0.05 mg (37 °C).	A 3.8-fold higher CA production by *A. pallidus* than that under unoptimized conditions. Enzyme thermostable that retains activity at alkaline pH: it is useful in carbon sequestration.	The partially purified enzyme produces precipitation of 42.5-mg CO_3_^2−^ mg^−1^ protein. CO_2_ sequestration is efficient.	[200]
	Study of the biochemical properties, thermostability, and inhibition of CA from *Sulfurihydrogenibium azorense*, SazCA.	Hepes buffer 10 mM, NaBF4 20 mM, pH 7.5. Phenol red (0.2 mM) as an indicator.	SazCA is highly thermostable and can survive incubation at 90–100 °C.	*k*_cat_ value 4.40 × 10^6^ s^−1^, *K*_M_ value 12.5 mM, *k*_cat_/*K*_M_ 3.5 × 10^8^ M^−1^ s^−1^, 5-fold faster than the second CA. SazCA is the second faster enzyme, after superoxide dismutase (SOD).	[201]
	To explore wollastonite (calcium silicate) carbonation for the removal of anthropogenic CO_2_ and evaluate the effectiveness of different natural (CA) and CA biomimetic catalysts (Zr-based MOFs) to enhance CO_2_ capture.	Water 170 mL, the catalyst (30 ppm), adjusted at pH 4 (25 °C). Wollastonite crystals (100 mg) added to the solution. CA is immobilized on the MOFs UiO-66 and MOF-808@Mg(OH)_2_ by an impregnation process.	CA accelerates carbonate precipitation but hinders carbonation of wollastonite. Thick carbonate coatings formed on the most reactive surfaces of wollastonite act as passivating layers leading to a reduction in the dissolution and carbonation rates.	The passivating effect explains why the conversion of wollastonite into calcite was so limited (up to 14 mol%). Zr-based MOFs accelerate the dissolution of wollastonite.	[202]

**Table 5:** CO_2_ reduction to obtain formate using whole-cell approaches.

Reduction	Organism	Significant Assay Features/Conditions	Formate Production	Ref
Hydrogenation	Overexpressed genes of FDH from *E. coli, Clostridium carboxidi-vorans, Pyrococcus furiosus* and *Methanobacterium thermos-formicicum* in *E. coli* JM109(DE3)	H_2_ atmosphere. Wet cell pellet (0.5 g wet cells/mL) resuspended in 50 mM sodium phosphate buffer, pH 7.0, containing 0.25 M sodium bicarbonate as a source of CO_2_. Incubation 37 °C.	The highest formic yield (FDH from *P. furiosus*) was more than 1 g L^−1^ h^−1^	[292]
Hydrogenation	*Escherichia coli*	CO_2_ and H_2_ placed under pressure (up to 10 bar). First, pressurized the system to rapidly convert 100% conversion of gaseous CO_2_ to formic acid. Next, NaOH addition to the *E. coli* cell suspension (pH > 8).	Formate concentration to 150 (4 bar) and 200 (6 bar) mmol L^−1^. Increasing the pressure to 10 bar (122.88 mmol L^−1^ CO_2_ and 3.61 mmol L^−1^ H_2_): > 0.5 mol L^−1^ formate at 23 h of reaction.	[293]
Hydrogenation	*Acetobacterium woodii* and *Thermoanaerobacter kivui*	Cells grown with 28 mM glucose or 0.1 M pyruvate (50 mM imidazole, 20 mM KCl, 20 mM MgSO_4_, 2 mM DTE, 4 μM resazurin, pH 7.0). 1 mg/mL in an anoxic medium. Shaking for 10 min (60 °C), with additional 300 mM KHCO_3_. The experiment started by replacing the gas phase with H_2_ + CO_2_ (80:20%, 2 × 10^5^ Pa).	Optimal formate production rates of 234 mmol g^−1^_protein_ h^−1^	[270]
Hydrogenation	H_2_-dependent CO_2_ reductase from *Acetobacterium woodie* expressed in *E. coli* JM109	Whole-cell E. coli in presence of formate (10 mM) and methylviologen (10 mM) as electron acceptor. *E. coli* cells incubated with H_2_ + CO_2_ (80:20%, 1.1 × 10^5^ Pa).	6 mM formic acid in 60 h. Addition of 2.5 mM HCO_3_^−^ increased 4-fold the formate generation.	[294]
Hydrogenation	*Acetobacterium woodii* and *Thermoanaerobacter kivui*	50 mM imidazole, 20 mM MgSO_4_, 20 mM KCl, 20 mM NaCl, 2 mM DTE, pH 7.0; or 50 mM K-phosphate, 20 mM KCl, 20 mM NaCl, 2 mM DTE, pH 7.0) was maintained at 30 and 60 °C for *A. woodii* and *T. kivui*, respectively. Gas flow rate was maintained at a value of 25 mL/min	60 mM and 80 formic acid generation after 5 h of reaction for *A. woodii* and *T. kivui*, respectively.	[295]
Electrochemical	*Methylobacterium extorquens* AM1,	System: 1 mM H_2_SO_4_ with a platinum electrode placed in the anode; protons supplied to the cathode through a proton-exchange membrane. 1.9 g wet cells, 10 mM methyl viologen (MV) added to the cathode reactor as an electron mediator	Formate concentrations of up to 60 mM, 80 h (1.9 g wet cells, 10 mM MV, pH 7.0)	[290]
Electrochemical	*Shewanella oneidensis* MR-1	The electrochemical cell with two compartments divided by a proton-exchange memberane. Copper plates and Ag/AgCl electrodes. Whole-cell *S. oneidensis* MR-1 (wet-cell, 0.5 g) and MV, 10 mM). RT, anaerobic conditions.	Formic acid at 0.59 mM h^−1^ for 24 h. Medium supplemented with fumarate and nitrate: 1.9 mM h^−1^ for 72 h. LB supplemented with 40 mM fumarate, 1mM nitrate and 20 mM DL-lactate: 136.84 mM formic acid at 72 h. (3.8 mM h^−1^ g^−1^_wet-cell_)	[296]
Electrochemical	*E. coli*	NaHCO_3_ electrolyte saturated with N_2_ or CO_2_ media at three different poised potentials, i.e., 0.4, 0.8 and 1.0 V vs. Ag/AgCl. *E. coli*-immobilized FePc-CDC/ACF and ACF electrodes in the presence of the NR mediator (FePc: iron pfthalocyanine; CDC: carbide-derived carbon; ACF: activated carbon fiber; NR: Neutral Red).	Maximum formate production rate of ~30 mg/L-h under CO_2_ flow (120 mg/L-h) with NR mediator	[297]
Electrochemical	*Methylorubrum extorquens* AM1	Nafion 115 (proton permeable) membrane. Cathode: 0.6 g cell pellet (potassium phosphate 200 mM, pH 7.0) as catholyte. Electron mediator: ethyl viologen 10 mM. 0.6 g cell pellet suspended into catholyte (200 mM-potassium phosphate at pH 7) saturated by CO_2_ purging (30 min). Water splitting reaction in 100 mM-H_2_SO_4_.	Formate production: 6 mM h^−1^	[298]
Electrochemical	*Shewanella loihica* PV-4	Cathode: biohydrogel formed by *Shewanella loihica* PV-4 immobilized in graphene oxide.	High Faradaic efficiency (~99.5%) and 46-fold increase of formate titer without exogenous mediator (4.2 mM formate. 36 h)	[299]
Hydrogen/car-bohydrate fermentation	*Saccharomyces cerevisiae*	Two phases: Glucose fermentation for generating CO_2_ CO_2_ Ru catalysis hydrogenation	26% of the CO_2_ was hydrogenated. Addition of His 150 mM: 128 mM in formic acid at 48 h.	[300]
Photocatalytic hydrogenation	*Shewanella oneidensis* MR-1	Anaerobic conditions: N_2_-filled chamber and samples irradiated from outside the chamber by a KL5125 Cold 150W light source. Irradiation into MV, with triethanolamine (TEOA) as sacrificial agent in 50 mM HEPES, 50 mM NaCl, pH 7, 23 °C.	Formate (∼1500 nmol) was produced when MR-1 was incubated with CO_2_ (∼5000 nmol) after 48 h incubation	[301]
